# Higher HIV-1 evolutionary rate is associated with cytotoxic T lymphocyte escape mutations in infants

**DOI:** 10.1128/jvi.00072-24

**Published:** 2024-05-30

**Authors:** Jamirah Nazziwa, Sophie M. Andrews, Mimi M. Hou, Christian A. W. Bruhn, Miguel A. Garcia-Knight, Jennifer Slyker, Sarah Hill, Barbara Lohman Payne, Dorothy Moringas, Philippe Lemey, Grace John-Stewart, Sarah L. Rowland-Jones, Joakim Esbjörnsson

**Affiliations:** 1 Department of Translational Medicine, Lund University, Lund, Sweden; 2 Lund University Virus Centre, Lund University, Lund, Sweden; 3 Nuffield Department of Clinical Medicine, University of Oxford, Oxford, United Kingdom; 4 Department of Microbiology and Immunology, University of California San Francisco, San Francisco, California, USA; 5 Department of Global Health, University of Washington, Seattle, Washington, USA; 6 Department of Epidemiology, University of Washington, Seattle, Washington, USA; 7 Department of Pathobiology and Population Sciences, Royal Veterinary College, London, United Kingdom; 8 Department of Paediatrics and Child Health, University of Nairobi, Nairobi, Kenya; 9 Department of Medicine, University of Washington, Seattle, Washington, USA; 10 Department of Microbiology, Immunology and Transplantation, Rega Institute, KU Leuven, Leuven, Belgium; 11 Department of Pediatrics, University of Washington, Seattle, Washington, USA; 12 Global Center for Integrated Health of Women, Adolescents and Children (Global WACh), University of Washington, Seattle, Washington, USA; The Ohio State University, Columbus, Ohio, USA

**Keywords:** HIV-1, vertical transmission, infant, disease progression, CTL responses, intra-host evolution

## Abstract

Escape from cytotoxic T lymphocyte (CTL) responses toward HIV-1 Gag and Nef has been associated with reduced control of HIV-1 replication in adults. However, less is known about CTL-driven immune selection in infants as longitudinal studies of infants are limited. Here, 1,210 *gag* and 1,264 *nef* sequences longitudinally collected within 15 months after birth from 14 HIV-1 perinatally infected infants and their mothers were analyzed. The number of transmitted founder (T/F) viruses and associations between virus evolution, selection, CTL escape, and disease progression were determined. The analyses indicated that a paraphyletic-monophyletic relationship between the mother-infant sequences was common (80%), and that the HIV-1 infection was established by a single T/F virus in 10 of the 12 analyzed infants (83%). Furthermore, most HIV-1 CTL escape mutations among infants were transmitted from the mothers and did not revert during the first year of infection. Still, immune-driven selection was observed at approximately 3 months after HIV-1 infection in infants. Moreover, virus populations with CTL escape mutations in *gag* evolved faster than those without, independently of disease progression rate. These findings expand the current knowledge of HIV-1 transmission, evolution, and CTL escape in infant HIV-1 infection and are relevant for the development of immune-directed interventions in infants.

**IMPORTANCE:**

Despite increased coverage in antiretroviral therapy for the prevention of perinatal transmission, paediatric HIV-1 infection remains a significant public health concern, especially in areas of high HIV-1 prevalence. Understanding HIV-1 transmission and the subsequent virus adaptation from the mother to the infant’s host environment, as well as the viral factors that affect disease outcome, is important for the development of early immune-directed interventions for infants. This study advances our understanding of vertical HIV-1 transmission, and how infant immune selection pressure is shaping the intra-host evolutionary dynamics of HIV-1.

## INTRODUCTION

The implementation of perinatal transmission prevention programs has significantly decreased the incidence of HIV-1 infection among infants on a global scale, resulting in an estimated prevention of around 1.4 million pediatric infections between 2010 and 2018 (1). Despite this success, approximately 150,000 children (0–14 years old) were still estimated to have acquired HIV-1 in 2020. In total, children account for 5% of the global population living with HIV-1 and 15% of all people who have died from AIDS-related diseases (2, 3). The progression of HIV-1 infection in infants typically follows a natural course that is distinct from that in adults. This can depend on the mode of transmission (e.g., *in utero, peripartum*, or *postpartum*) or maternal intrinsic factors such as blood plasma viral load (VL) and human leukocyte antigen (HLA) genotype (4, 5). Progression to AIDS is also faster among infants, where approximately half of antiretroviral therapy (ART)-naïve infants progress to AIDS within the first year of infection, compared to approximately 7% in adults (6–9). The VL in perinatally infected infants remains elevated during the first year of infection, which contrasts the rapid decline in viremia typically observed in adults (10). A possible explanation for this may be that infants have a more limited and functionally ineffective CD8+ T cell response to HIV-1 (5, 11). Additionally, HIV-1 can undergo mutations that allow it to evade cytotoxic T lymphocytes (CTLs). These variants can then be transmitted from the mother to the infant and compromise the infant’s ability to mount specific immune responses due to pre-adaptation of the virus to the shared maternal HLA alleles (12–14). Currenti et al. recently showed that post-transmission adaptation occur in HIV-1-infected children aged 2–8 years, indicating selection in HIV-1 epitopes targeted by the T cell responses in this population (15). However, it is still unclear if, and to what extent, selection of HIV-1 CTL escape variants occurs in infants, and how this relates to infant disease progression.

In a previous study, we showed that infant CTLs can exert selective pressure on Gag and Nef epitopes during early HIV-1 infection (16). However, this study was based on bulk sequences that were not matched to the corresponding maternal samples. The intra-host transmission dynamics of CTL variations between the mother and infant was therefore not addressed in that study.

Several other studies have also investigated the genetic bottleneck that occurs following vertical and horizontal transmission of HIV-1, focusing on the env region (17–24). However, variations have been observed in the frequency of single transmitted variants among different cohorts of infants. These differences may arise from variations in sample collection timing after transmission, analysis methodologies, and the number of patients studied. Gaining a comprehensive understanding of the transmission event, along with the characteristics and evolution of transmitted/founder (T/F) viruses in response to the host immune system in infants, is crucial for the development of early immune-based interventions and vaccines targeted at infants. In this particular study, clonal sequences of HIV-1 obtained from a historical cohort of mother-infant pairs were utilized to achieve two main objectives: (i) to determine the intra-host diversity and evolutionary patterns of the *gag* and *nef* regions during the first 15 months of life in infants with different rates of disease progression, utilizing advanced phylogenetic approaches, and (ii) to characterize the transmission dynamics of CD8+ T cell escape variants from mother to infant.

## RESULTS

### Cohort and participant characteristics

Data and samples were obtained from a cohort of 465 women living with HIV-1 in Kenya (25). Of these, 72 gave birth to babies who acquired HIV-1 within the first month of life, and *in utero* or perinatal infection was determined as described previously (25). The women were followed from 1999 to 2005, with infants followed for approximately 2 years after birth (please see Materials and Methods for details). Longitudinal samples collected within the first 15 months of life from three or more time points were available for 23 of the infants. Mothers received short-course zidovudine (ZDV) in the final trimester of pregnancy to reduce the risk of vertical HIV-1 transmission, whereas infants did not receive treatment during follow-up due to national infant ART guidelines at the time.

In total, 1,210 *gag* and 1,264 *nef* sequences were successfully generated from 14 of the 23 infants and their mothers (Fig. S1A). Reasons for why sequences could not be generated for all time points and mother-infant pairs were sample not being collected, depleted sample, or failed PCR or sequencing. Eight of the 14 infants were males (Table 1). Four of the 14 infants were infected *in utero*, and 10 were HIV-1 negative at birth, but acquired infection before 1 month of age. Twelve infants were infected with subtype A1 viruses (as confirmed in both *gag* and *nef*), and two infants were infected with recombinants, as previously shown (Table 1) (16).

**TABLE 1:** Characteristics of the study participants^*a*^

ID	Sex	TM	BF	CD4%	PeakVL	Gag	Nef	Died <2 yrs	#*gag* TP	#*nef* TP	HLA-A	HLA-B	HLA-C	Prog	CTL escape
135	M	IU	Yes	10	9,011,700	A1	A1	Yes	3	3	32:6802	39:44	4:12	Fast	Yes
159	F	P	No	26	614,400	A1	A1	No	2	4	29:30	4501:4501	6:6	Slow	No
168	M	P	Yes	22	40,671,500	A1	A1	Yes	4	4	24:29	15:5802	2:4	Fast	No
170	M	IU	No	21	174,700	A1	A1	No	3	4	30:3402	4201/2:57	7:17	Fast	Yes
211	M	P	Yes	18	2,978,300	CD	CD	No	5	4	30:6802	4201/2:4201/2	7:17	Slow	Yes
231	F	P	Yes	22	1,152,900	AD	A1	No	4	4	26:34	35:53	4:4	Slow	No
258	M	IU	No	25	3,872,900	A1	A1	No	3	3	3:30	15:7301	15:17	Slow	Yes
259	M	P	Yes	34	1,038,100	A1	–	No	2	0	23:74	58:4501	7:7	Slow	No
261	F	P	Yes	28	524,000	A1	A1	No	4	5	2:29	5802:35	6:7	Slow	No
281	M	P	Yes	25	211,000	A1	A1	No	4	4	23:74	15:15	2:2	Slow	No
291	M	P	No	14	7,047,400	A1	A1	No	4	6	29:74	4201/2:15	2:17	Fast	No
334	F	P	Yes	26	1,613,200	A1	A1	Yes	4	4	29:26/66	13:15	2:6	Slow	Yes
411	F	P	Yes	16	3,068,000	A1	A1	No	3	3	30:30	15:42	14:17	Fast	Yes
424	F	IU	Yes	7	N/A	–	A1	Yes	0	3	2:6802	51:8	7:1601	Fast	NA

^*a*^ ID, patient identification number; sex (M, male; F, female); TM, mode of HIV-1 transmission (IU, *in utero*; P, *peripartum*); BF, breastfeeding status; CD4% (percentage of CD4+ T cells) at month 6; PeakVL, maximum viral load measured within 6 months of infection in copies per milliliter of plasma; *gag*, HIV-1 subtype as determined by *gag* genotyping; *nef*, HIV-1 subtype as determined by *nef* genotyping; died <2yrs: infants that passed away before their second year of age; #*gag* TP, number of time points analyzed for *gag*; #*nef* TP, number of time points analyzed for *nef*; HLA-A, human leukocyte antigen class A allele; HLA-B, human leukocyte antigen class B allele; HLA-C, human leukocyte antigen class C allele; Prog, HIV-1 disease progression status; CTL escape, presence of *gag* cytotoxic T cell lymphocyte (CTL) epitope escape mutations; NA, not applicable. The presented ELISPOT data were generated in a previous study (11).

**Fig 1:**
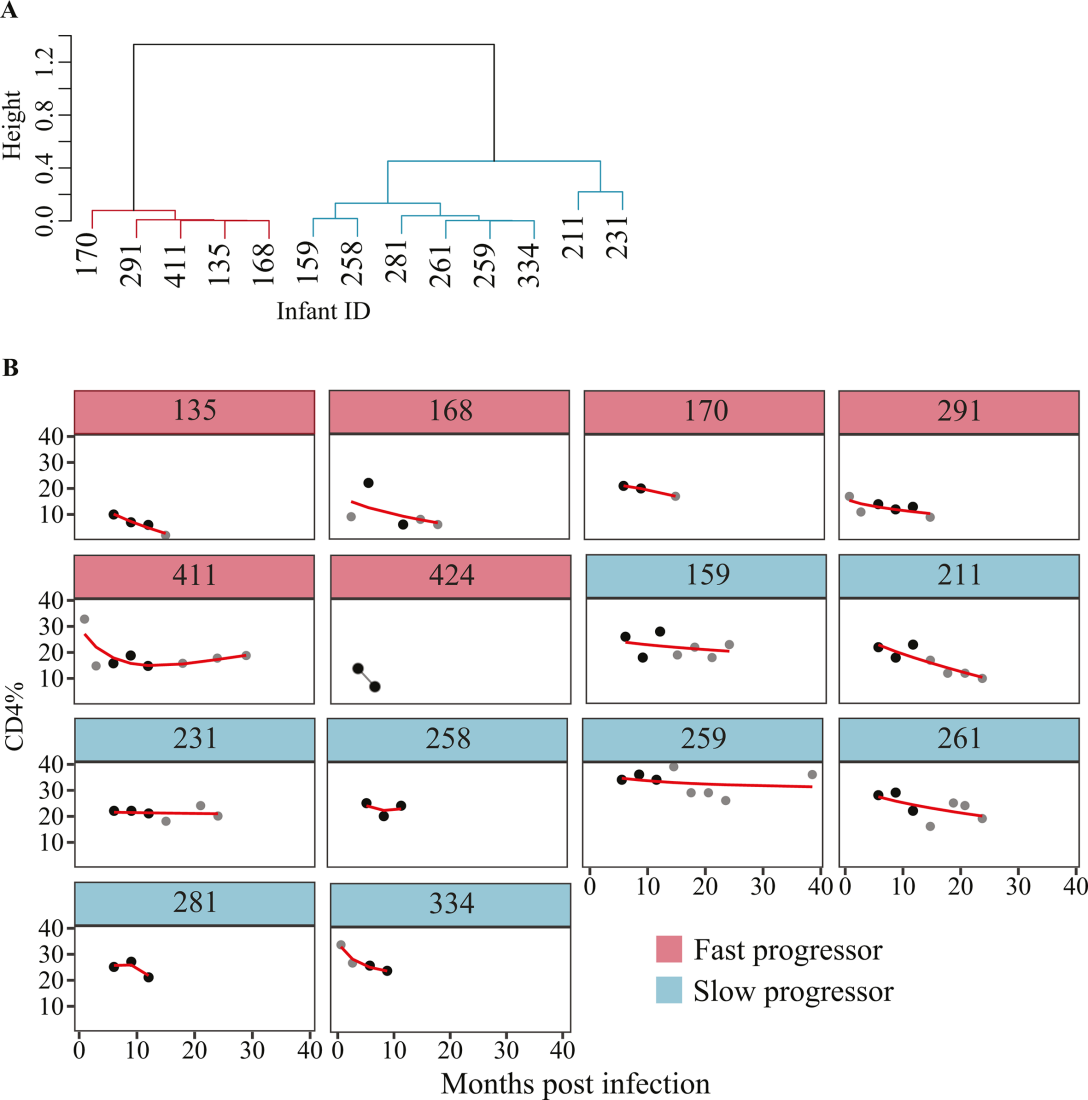
Classification of infant disease progression status based on CD4% decline rate. (**A**) Dendrogram representing the hierarchical clustering pattern of infants based on the CD4% data collected at the time points. Red cluster, fast progressors; blue cluster, slow progressors. Infant 424 had less than three CD4% measurement and was excluded from clustering analysis because of insufficient unique time values to support the cubic smoothing. (**B**) Line plots showing infant CD4% dynamics over time. Red line indicates the predicted CD4% values from the generalized additive models (GAM) that were used in the hierarchical clustering. The black dots indicate the time points with CD4% data that were used in the analysis and no extrapolation was used. The gray data points represent those time points with CD4% data but were not included in the phylogenetic analyses.

The median CD4% 6 months after birth was 22.0% (IQR: 16.5%–22.8%), and the median plasma VL was 11.4 million copies/mL (IQR: 3.7–25.4 million copies/mL) among the 14 studied infants. The median maternal plasma VL was 0.3 million copies/mL (IQR: 0.1–0.6 million copies/mL, Fig. S1B), and was significantly lower than the infant median VL (*P* < 0.001, Wilcoxon rank-sum test). Four of the 14 infants died before 2 years of age, and two of these four infants acquired HIV-1 during birth (Table 1). We investigated disease progression by analyzing the longitudinal decline in CD4% levels. Out of the 14 infants studied, six were classified as fast progressors, as their CD4% remained below 20% for up to 20 months or if they were alive. Three of these six infants passed away before reaching the age of 2 (Table 1; Fig. 1). The remaining eight infants were classified as slow progressors as their CD4% remained above 20%.

### Most infants were infected by a single transmitted founder virus

**Fig 2:**
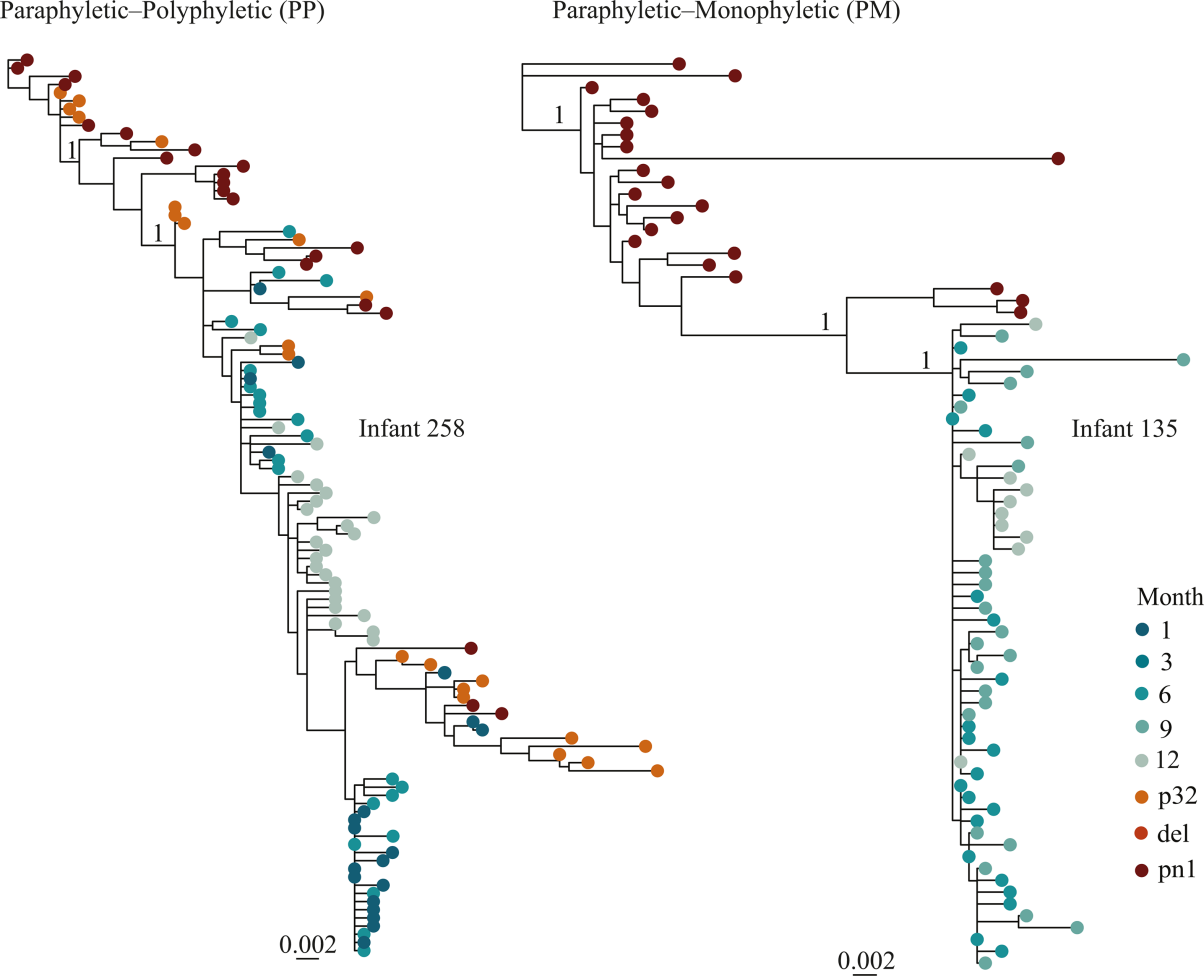
Phylogenetic tree topology classes (based on *gag* sequences) between mother and infant transmission pairs. Maximum-likelihood phylogenetic trees for two paired mother and infant sequences exemplifying the two different tree topologies observed. The red tips represent mother sequences whereas the green tips represent sequences of the paired infant. In the PP class, the infant sequences were nested in more than one cluster among the mother sequences (infant 258). In the PM class, the infant sequences were nested in one cluster among the mother sequences (infant 135). Relevant branches supported by an approximate likelihood ratio test (aLRT-SH) score of 1 are indicated by “1” in the trees. The scale bar represents substitutions per site. All mother-to-child-transmission trees are presented in Fig. S2. Abbreviations: p32, 32 weeks of pregnancy; del, delivery; pn1, 1 month after delivery.

We confirmed mother-to-child HIV-1 transmission based on maximum likelihood (ML) phylogenetic constructions with *gag* and *nef* sequences from the infant and mother samples. *Gag* and *nef* sequences could not be generated from five and nine mothers, respectively (Fig. S1A). The number of T/F viruses from an average of 23 *gag* or *nef* sequences collected per infant 1 month after birth was quantified using inspection of alignments in LANL highlighter plots, neighbor-joining trees, Hamming distances, and genetic diversity as described by Keele et al. (17). Ten of the 14 infants had sequences collected 1 month after birth, which were utilized for the T/F analysis. The remaining five infants were excluded from the analysis to prevent potential biases associated with time-related variations in diversity estimates. Two of the nine infants (22%) were infected with more than one T/F virus (Table S1; Fig. S2). Visual inspection of the alignment highlighter plots indicated that sequences from these two infants formed more than two distinct lineages, characterized by at least three nucleotide polymorphisms shared among multiple clonal sequences. Recombination events between two lineages were observed in both infants with multiple T/F viruses (Fig. S2B and D). Furthermore, the mean *gag* and *nef* diversity increased over time (*P* < 0.001, Kruskal-Wallis H test, Fig. S3). The mean diversity between *nef* [0.0073 substitutions per site (95% CI: 0.0070–0.0076 substitutions per site)] and *gag* [0.0056 substitutions per site (95% CI: 0.0054–0.0057 substitutions per site)] was not significantly different (*P* = 0.14, Wilcoxon rank-sum test Fig. S4). Next, we generated ML trees including both the mother (donor) and the infant (recipient) sequences for each pair separately (*gag* and *nef* sequences, respectively). The resulting phylogenies could be divided into two groups: (i) paraphyletic-polyphyletic (PP, where infant sequences formed several clusters, interspersed among the maternal sequences, indicating presence of multiple T/F viruses); and (ii) paraphyletic-monophyletic (PM, where infant sequences nested within the mother sequences in one cluster and indicated a single transmitted/founder variant in the infant (Fig. 2; Fig. S5) (26). Nine of the 14 infants had both mother and infant sequences in *gag*, while five pairs had sequences in *nef* and were included in the analysis (Fig. S1A). Seven of the nine mother-infant pairs had PM tree topologies, whereas two of the pairs had PP tree topologies in *gag* (Fig. 2; Table S2). All the five mother-infant pairs with *nef* sequences had PM tree topologies (Fig. 2; Table S2). Except for infant 231, all infants associated with PM tree topologies corresponded with a single T/F virus as determined by the Keele method (Table S2). Moreover, all infants that were associated with PP tree topologies corresponded with multiple T/F infections as determined by the Keele method (Fig. 2; Fig. S3) (17). Taken together, the analyses suggested that HIV-1 infection was established by a single T/F virus in 10 of the 12 infants (83%).

### Selection occurs from 3 months and onward during infant HIV-1 infection

**Fig 3:**
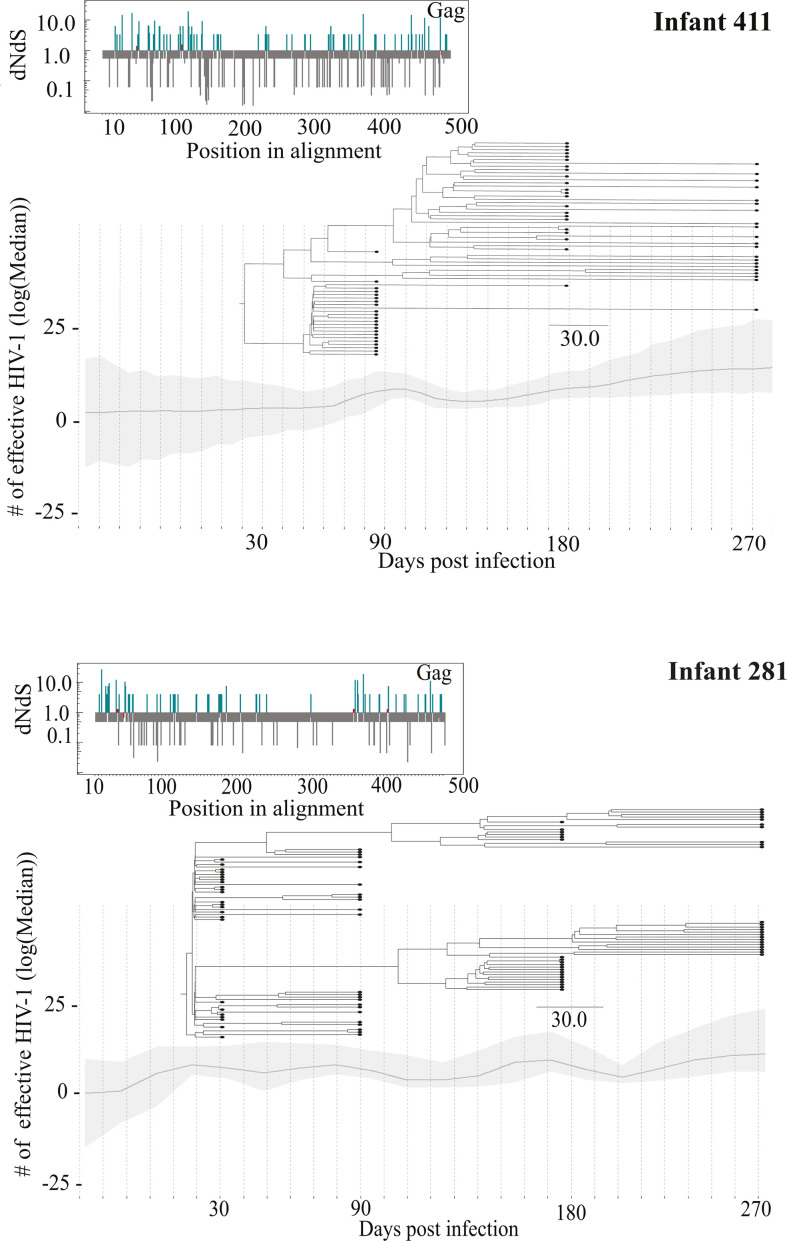
Intra-host HIV-1 evolutionary dynamics. Infant 411 showed no selective sweeps, and infant 281 showed evidence of selection. The line graph indicates the number of effective HIV-1 on log scale over time. A maximum clade credibility phylogenetic tree was layered over the graph to indicate how the virus evolved within the patient over time. The insert on the left shows amino acid sites under positive and negative selection in Gag. Blue lines, sites under positive selection; gray lines, sites under negative selection; red line, sites under neutral selection.

Bayesian phylodynamics were used to assess how the evolutionary dynamics of the transmitted viruses from the mother evolve within the infants. Initial analysis of the temporal signal in each data set indicated a moderate (correlation coefficient >0.5) to fairly strong (correlation coefficient >0.8) signal in 75% of *gag* and 62% of *nef* infant data sets. However, even with a moderate phylogenetic signal and several attempts to optimize model settings, the phylodynamic modeling failed to converge in four of the *gag* data sets (infants 159, 168, 259, and 334) and in four of the *nef* data sets (infants 159, 261, 334, and 424, Fig. S6). For the sequences that converged, one or multiple selective sweeps (i.e., high frequency of genetic variants observed due to adaptation) were observed in both *gag* and *nef* based on a maximum clade credibility tree structure (Fig. 3; Fig. S6). In *gag*, selection of some virus populations was observed already 3 months post-transmission (infant 231), whereas most infants displayed selective sweeps from 6 months and onward (Fig. S6A). Analysis of infant 258 (who was infected by multiple T/F viruses) indicated that two virus populations co-existed up until 6 months of age, after which one virus population became dominant. Assessment of the amino acid alignments showed that nonsynonymous changes occurred in CTL epitope positions among infants 135, 170, 211, 258, and 334, indicating immune-driven selection pressure (Table S3). In *nef*, selective sweeps were observed at 3 months post-transmission in infants 168, 231, 281, and 291, and multiple sites were under positive selection (Fig. S6B). In addition, multiple virus populations co-existed and co-evolved in infants 231 and 258 (infected with multiple T/F viruses) also after 3 months of age (Fig. S6B).

### Presence of HIV-1 CTL escape variants in 7 of 13 infants

To elucidate the impact of immune-driven selection pressure associated with CTL escape mutations, a comparative analysis between mother and infant sequences was done with a specific focus on HLA-restricted epitope positions in Gag and Nef. Twenty-three epitopes from 11 infants were examined in Gag. The renaissance counting analysis indicated that at least one amino acid was under positive selection in 17 of these 23 epitopes (Table S5A). Moreover, 16 of the 23 epitopes could be further assessed in nine mother-infant pairs showing that escape variants were transmitted and consistently present within the mother-infant pairs across all time points for 14 out of the 16 epitopes (88%, Tables S5 and S6). Among the seven infants assessed, four (258, 411, 135 and 334) had paired data with their respective mothers (Table 2). In 14 of the CTL epitopes examined in the mother-infant pairs, escape variants were observed in both maternal and infant sequences. However, for 2 of the 16 Gag epitopes (HXB2 positions 145–155 and 429–437), CTL escape variants were observed in infant 334 at 6 months of age, but not in the corresponding maternal sequences (Table 2). Interestingly, these escape variants were not detected in the sequences from earlier time points, or in the corresponding maternal sequences, suggesting that post-transmission adaptation occurred in this infant. In the Nef region, five epitopes from four infants were analyzed, in which at least one amino acid was under positive selection in four of the epitopes (Table S5B). Two of these could be further assessed in five mother-infant pairs, and the analysis showed that the escape variants were consistently transmitted and maintained across all time points (Table S6).

**TABLE 2:** Infant CTL escape variants in the targeted Gag and Nef epitopes^*a*^

ID	HLA	HXB2 position	Epitope sequence isolated from infant and/or mother	Mutation frequency in infant	Time point in months (infant)	Mutation frequency in mother	Time point in months (mother)	ELISPOT wild-type peptide stimulation results (infant)
		Gag						
		18–26	KIRLRPGGK					
258 + M	A3		R--------	16/20, 11/22, 1/24	1,6,12	10/22,2/21	Del, pn1	(−) 3,6,12
		20–29	RLRPGGKKKY					
411 + M	B15		--------Q-	21/22, 21/22, 12/21	3,6,9	18/21	Del	NA
		76–86	RSLYNTVATLY					
135 + M	A68		K--F-A--V--	20/20, 24/24, 10/10	6,9,12	3/21	pn1	NA
170	A30		K----------	19/19, 14/22, 5/24	6,9,12	–		(−) 3,6,9,12
211	A30		K-------V--	21/21,11/22, 23/23, 19/19, 23/23	3,6,9,12,15	–		(−) 3,6,9,12
411 + M	A30		K--F-A--V-F	22/22, 22/22, 21/21	3,6,9	21/21	Del	(−)1,3,6,9,12
		145–155	QAISPRTLNAW					
334 + M	C6		--M--------	0/21, 0/18, 21/21, 23/23	1,3,6,9	Not observed	Del	NA
		167–175	EVIPMFSAL					
334 + M	A26		------T--	21/21, 18/18, 21/21, 23/23	1,3,6,9	19/19	Del	NA
		180–188	TPQDLNTML					
211	B42		--G------	21/21, 22/22, 23/23, 3/19	3,6,9,12	–	NA	(−) 3,6
211	B42		--A------	14/19	12	–	NA	(+) 12
211	B42		--T------	2/19, 23/23	12,15	–	NA	(+) 12
		272–285	YSPTSILDI					
135 + M	C12		---V-----	20/20, 24/24, 10/10	6,9,12	21/21	pn1	NA
		294–304	DYVDRFYKT					
334 + M	A26		------F-I	21/21, 18/18, 21/21, 23/23	1,3,6,9	19/19	Del	(−) 3,6,9
		385–393	GPKRIVKCF					
211	B42			4/21, 11/22, 17/23, 12/19, 7/23	3,6,9,12,15	–	NA	NA
211	B42		-TR--I---	2/21, 3/19	6,12	–	NA	
211	B42		--R------	7/21, 7/22, 2/23, 8/23	3,6,9,15	–	NA	NA
211	B42		--L--I---	4/21	3	–	NA	NA
211	B42		--R-M----	8/23	15	–	NA	NA
211	B42		--L--I---	5/21	3	–	NA	NA
		429–437	RQANFLGKI					
334	B13		--------L	21/21, 18/18, 2/21, 20/23	1,3,6,9	Not observed	Del	NA
		Nef						
		77–85	RPMTFKGAF					
170	A30		S---Y-A-I	12/24, 24/24l, 10/22, 7/12	3,6,9,12	–	NA	NA
170	A30		S---Y-A-V	12/24, 3/22, 3/12	3,9,12	–	NA	NA
170	A30		S---Y-A-M	5/22, 2/12	9,12	–	NA	NA
211	B42			3/20, 23/23	9,15	–	NA	NA
211	B42		----Y-A-F	23/23, 17/22	6,12	–	NA	NA
211	B42		---NY-A-V	17/20, 5/22	9,12	–	NA	NA
		83–91	AAVDLSHFL					
424	A6802		G-L------	22/22, 24/24, 12/24	1,3,6	–	NA	NA
		120–128	YFPDWQNYT					
334 + M	B13		-----H---	24/24, 10/21	1,3	24/24	P32	NA
334	B13		-----O---	11/21, 20/20, 23/23	3,6,9	Not observed	P32	NA

^*a*^ HXB2 position, gene-specific positions, i.e., Gag and Nef separately; epitope sequences, HBX2 epitope sequences and the alignment of patient sequences in the respective epitope positions; ID, patient IDs and + M indicate the same mutation was observed in the mother sequences, if available; mutation frequency in infant, the number of infant sequences where the mutation was present for each time point in months separated by comma; frequency in mother, the number of mother sequences where the mutation was present for each time point (P32, pregnant at 32 weeks; Del, delivery; pn1, 1 month after delivery; NA, no mother sequences available), separated by comma; ELISPOT, (NA, ELISPOT assay not done; (−) negative ELISPOT observed at different months separated by comma; (+) positive ELISPOT observed at different months separated by comma).

ELISPOT data on transmitted and acquired escape epitopes had previously been generated from the cohort by Lohman et al. (11). In brief, 27 Gag and 13 Nef peptides were used based on the common HLA class 1 alleles and subtype variants representative of East African populations, and the results were dichotomized as negative or positive, as previously described (Table 2) (11). The analysis of infant 211 indicated a dominant HLA-B42 restricted response from month 12 (no response was seen at months 3 and 6) when tested for the Gag epitope TPQDLNTML (Gag: 180–188). Sequence analysis showed that this infant had a mutation in the HXB2 Gag position 182, and that the glutamine in this epitope had changed to glycine in all the sampled sequences during early follow-up (months 3, 6, and 9). However, at month 12, 16 of 19 (73%) sequences had alanine at this position that later changed to threonine in 22 of 23 sequences at month 15. Mutation to glycine was not recognized by CTL, whereas mutations to alanine and threonine were associated with a positive IFN-γ response (Table 2). The escape variants observed in other infants led to undetectable CD8+ T cell responses (i.e., <100 spot-forming units per million peripheral blood mononuclear cells). Overall, emergence of CTL escape was observed in only one infant.

### CTL escape mutations in Gag were associated with higher evolutionary rates, but not disease progression

**Fig 4:**
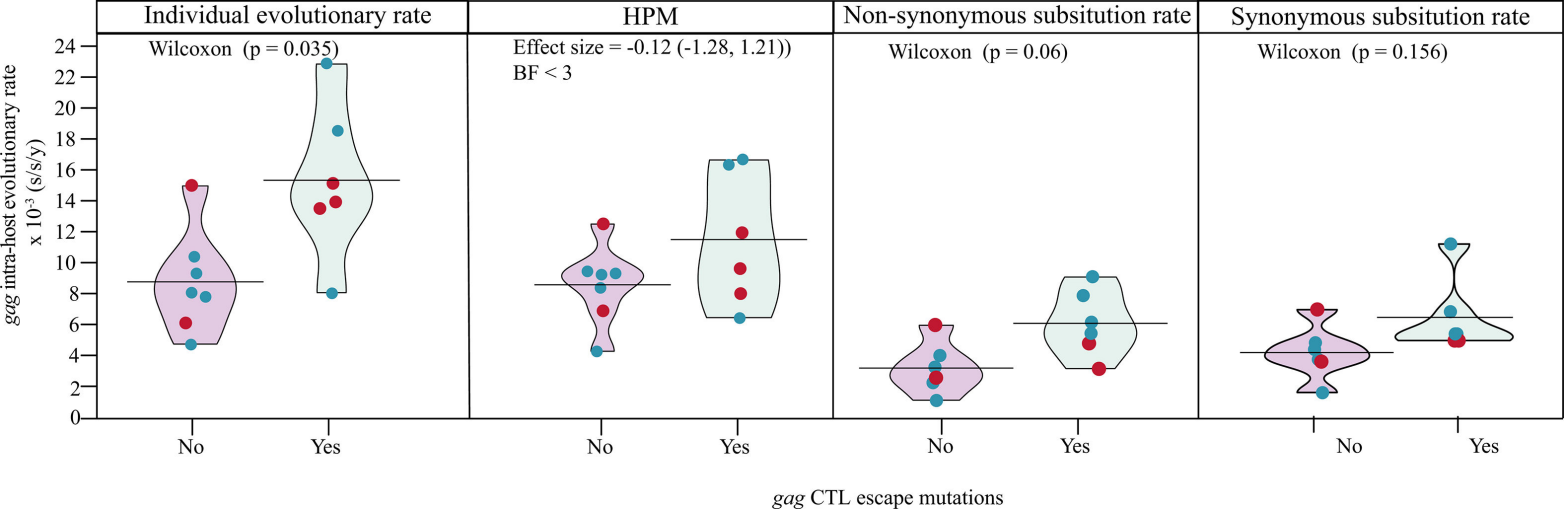
Pirate plots of the individual and HPM mean *gag* intra-host evolutionary rate in infants with and without CTL escape variants. Red points, fast progressors; blue points, slow progressors.

We next investigated the association between disease progression rate and intra-host HIV-1 evolutionary dynamics. When analyzed for each study participant independently, the intra-host HIV-1 evolutionary rate estimates were higher in *nef* compared with *gag* (*P* < 0.001, Wilcoxon rank-sum test, Fig. S7). However, to assess the evolutionary rate estimates more efficiently between groups, we employed a hierarchical phylogenetic model (HPM). The HPM has been shown to effectively shrink the variance of estimated parameters, as previously described (27). The analysis showed that the evolutionary rate was significantly higher in *nef* compared with *gag* (*P* < 0.001, Wilcoxon rank-sum test, Fig. S8). In addition, and in contrast to *gag*, the HPM indicated that the evolutionary rate was relatively similar across study participants in *nef* (Fig. S8). To further disentangle the association between disease progression rate and intra-host HIV-1 evolutionary dynamics, we therefore focused the subsequent evolutionary rate analyses on *gag*. Six of the 13 infants had CTL escape variants in *gag*, whereas the remaining did not have escape variants. The mean individual *gag* evolutionary rate was higher in infants with CTL escape variants compared to those without (*P* = 0.035, Wilcoxon rank-sum test, Fig. 4). However, the significant difference was not verified in the HPM with fixed effects [posterior probability mean for escape effect = 0.39; posterior effect size = −0.12 (−1.28, 1.21), Bayes factor <3, Fig. 4)]. In addition, no significant difference was observed in median *gag* evolutionary rate between fast and slow progressors (Wilcoxon rank-sum test, *P* = 0.583). To further disentangle the molecular adaptation process, we analyzed the expected nonsynonymous (E[N]) and synonymous (E[S]) substitution rates, reflecting the respective contribution of E[N] and E[S] substitution rates to the overall substitution rate (28). No significant differences in *gag* E[N] and E[S] substitution rates were found between infants with fast vs slow disease progression.

## DISCUSSION

In this study, we determined the intra-host diversity and evolution of *gag* and *nef* during the first 15 months of HIV-1 infection in a cohort of perinatally infected infants with different rates of disease progression. Previous reports have suggested low level of diversification and genetic diversity in HIV-1 subtype B and C *nef* and *gag* sequences in infants following transmission (13, 29, 30). Previous reports have also suggested that multiple T/F viruses from mother to child can be transmitted (18, 31–35). In this study, we observed that multiple T/F viruses occurred at a relatively similar frequency in infants compared with what has been observed following heterosexual transmission among adults (17, 21). Other studies of adult HIV-1 transmission have suggested that different phylogenetic topologies, reflecting the relationship between donor and recipient virus populations, can be utilized to assess the number of T/F viruses (36, 37). This may be particularly useful if samples collected very early in infection are not available. In line with this, our analysis showed similar relationships, suggesting that the number of T/F viruses can be assessed with high likelihood based on the tree topology between donor and recipient virus populations also in HIV-1 mother-to-child transmission.

On a more specific level, multiple variants were also observed in an infant infected *in utero*, suggesting that transmission of multiple T/F viruses can occur in this mode of vertical HIV-1 transmission. However, considering that the infant was infected several weeks before birth, it is also possible that the analyzed sample was collected too far from infection to properly assess the number of T/F viruses [samples should preferably be collected before CTL-driven immune selection contributes to the virus diversification, as suggested by Keele et al. (17)]. Either way, the probability of multiple T/F viruses related to *in utero* infection will need to be assessed in a larger data set. Moreover, conflicting results in the number of T/F viruses were observed in one infant (infant 231), suggesting multiple T/F viruses in the *nef*, but not in the *gag* analysis. Notably, infant 231 had a relatively diverse founding population compared with infants determined to have been infected by a single T/F virus. Moreover, putative recombinants were also identified within the first month of infection, highlighting the importance of disentangling the role of recombination in quantifying the number of T/F viruses. This can partly be assessed through full genome sequencing. In support, a review by Baxter et al. reported that studies using smaller HIV-1 fragments tended to overestimate the number of T/F viruses compared to studies based on near full-length HIV-1 genomes (21). It is also possible that other factors, like the mother’s viral load, clinical stage, antiretroviral treatment status, and presence of sexually transmitted infections can influence the number of transmitted viruses.

Several studies have indicated the persistence of maternal CD8+ T cell escape variants transmitted from mothers to their infants (13, 14, 29). In general, similar patterns were observed in our study, suggesting that these escape variants do not significantly limit the HIV-1 fitness when transmitted to the child. However, a few epitope escape variants developed within infant 334 from 6 and 9 months after birth that were not observed in the mother. We lacked HLA information on the mother, we could not determine whether the virus was passing from a non-selective immune environment (HLA dissimilar in child and mother). It is also possible that this could be due to selective pressure restricted by an HLA allele inherited from the father. Frequent transmission of Gag epitope escape mutants has also been reported in adults, whereas escape in infants mainly occurred in epitopes restricted by maternal HLA alleles, implying escape from maternal CD8+ T cells (13, 38). Taken together, our analysis, focusing on infants under 2 years of age, complements work by Currenti et al. on children aged 2–8 years (15). This suggests that CD8+ T cell responses in early infancy may exert selective pressures on the virus population, both in older children and as early as 3 months of life. In addition, selection of some virus populations was already observed 3 to 6 months post-transmission, and in cases where two virus populations co-existed, one typically outcompeted the other over time. Although the capacity of the CD8+ T cell response to eliminate HIV-1 remains to be fully quantified, our results support the notion that early immune-directed interventions could improve the efficacy of CTL responses in infants infected with HIV-1.

The finding that HIV-1 populations harboring CTL escape mutations in *gag* evolved faster than those without may have several explanations. For example, it is possible that virus variants able to escape, or partially escape, the CTL responses are being cleared in a less effective way, resulting in a fitness advantage compared to virus populations not carrying these escape mutations. Moreover, the evolutionary rate is determined by both synonymous and nonsynonymous substitutions, implying that the viruses with CTL escape mutations in *gag* also replicate faster. However, this remains to be confirmed in future studies, e.g., comparing the replicative capacity of viruses with and without CTL escape mutations as previously done in adult acute HIV-1 infection (39). Despite some male-female imbalance in our study (8 of the 14 study participants were male), the results are in line with previous research indicating that the homogeneity in early virus populations is independent of sex. Our results also support the perception that sex differences associated with HIV-1 disease progression may not be linked to HIV-1 evolution (40).

In summary, this study provide novel insights into the diversity and evolutionary dynamics of the HIV-1 *gag* and *nef* regions during the early stages of pediatric HIV-1 infection. The results are relevant for future research on early immune-directed interventions and the efficacy of CTL responses in HIV-1 infection among infants, with key conclusions being (i) multiple T/F viruses were present in 22% of mother-to-child transmissions; (ii) CTL-mediated immune selection occurred as early as 3 months after birth in both *in utero* and perinatal infections; (iii) intra-host virus populations with CTL escape mutations in *gag* evolved faster than those without escape variants independent of the rate of disease progression; and (iv) specific HIV-1 subpopulations were selected in the majority of infants from 6 months of age and onward.

## MATERIALS AND METHODS

### Study subjects and ethical considerations

Data and samples were obtained from 14 Kenyan infants (≤2 years old) and their mothers who were part of a historical cohort from 1999 to 2005 (25). Infants were followed for up to 2 years after birth. Due to the lack of a national ART program and no guidelines on empiric infant ART until after 2008, infants in the cohort were treatment naïve. Mothers received short-course ZDV in the final trimester of pregnancy to reduce the risk of vertical HIV-1 transmission, but did not receive any treatment during breastfeeding or after delivery (41, 42). Infants were either infected *in utero*, *peripartum*, or through breastfeeding (43).

HIV-1 *gag* PCR was used to detect HIV-1 in infants using plasma or dried blood spots. Infant HIV-1 infection was categorized as *in utero* on detection of a positive *gag* PCR within the first 48 hours of life, or *peripartum* (*intrapartum*, or via early breastfeeding) if the *gag* PCR HIV-1 was negative or undetectable >48 hours after birth and <1 month of life (11, 43).

Blood plasma samples from infants were collected at birth, at 1 month, and then at 3, 6, 12, 15, 18, 21, and 24 months (11). Blood samples from the mothers were collected at week 32 of the pregnancy, at delivery, and 1 month postnatally. Infant and maternal VL, CD4+ T cell counts, and HLA types were determined by conventional protocols (16). For evolutionary analyses, infants were selected if they were infected by 1 month of age and had more than two longitudinal plasma specimens (16, 43).

### Amplification, cloning, and sequencing

Viral RNA was isolated from the plasma samples using the QIAamp Viral RNA extraction kit (Qiagen, Limburg, Netherlands) and amplification of the full *gag* (HXB2 positions 790–2289) and *nef* (HXB2 nucleotide positions 8797–9414) region was performed using in house PCR assays described elsewhere (16). Four microliters of purified *gag* or *nef* PCR product was combined with 1 μL of pCRTM4-TOPO plasmid stock from the TOPO-TA cloning kit (Invitrogen, Carlsbad, CA, USA) and 1 μL of salt solution, incubated for 30 minutes at room temperature, and then stored at −20°C until transformation. Five microliters of vector (PCR product ligated to plasmid) was added to one vial of chemically competent *E. coli* TOP10 cells from the TOPO-TA cloning kit (Invitrogen, Carlsbad, CA, USA) gently mixed, and incubated on ice for 30 min. The cells were then heat-shocked at 42°C for 30 seconds in a water bath. Cells were then rested on ice for 2 minutes. Two hundred fifty microliters of sterile Super Optimal broth with Catabolite repression media was added to each vial. Cells were then incubated at 37°C for 1 hour in a shaking incubator rotating at 225 rpm. After incubation, 75 µL of each vial was added to Luria-Bertani (LB) agar plates (10 g LB; 7.5 g agar; 500 mL water; 500 µL of 50 mg/mL kanamycin) and incubated for 16 h at 37°C. Vector-only negative controls were added to each experiment to exclude contamination between plates. Twenty-five clones were picked routinely and amplified in a colony PCR using the Advantage 2 PCR kit (Takara). In the colony PCR, we used the same primers as the nested reaction described elsewhere and the colony PCR products were then Sanger sequenced with the inner amplification primers at the Macrogen Europe sequencing facility (Amsterdam, Netherlands) (16).

### ELISPOT data

The ELISPOT data were generated in a previous study and the stimulation results were interpreted as either positive or negative (11). In brief [and as outlined in the study by Lohman et al. (11)], the following criteria were used to determine a positive assay: (i) a response to PHA of ≥100 SFU after the subtraction of the background, (ii) the number of HIV-specific SFU per 10^6^ cells being greater than or equal to 50, and (iii) the number of SFU in peptide-stimulated wells at least twice the number of background control SFU.

### Phylogenetic analysis

To confirm mother-to-child transmission, phylogenetic analysis was performed using the generated clonal *gag* and *nef* sequences from infants collected 1 month after delivery and the mother at week 32 of pregnancy or delivery or 1 month after delivery. Recombinant sequences were excluded from the analysis using the Phi test (Φ_w_) (44). The generated sequences were aligned per study participant using CLUSTAL W (45). ML phylogenetic trees were reconstructed using the inferred model, GTR +I +G, with GARLI v.2.01 (46). Statistical support for internal branches was determined by ML-based approximate likelihood ratio test (aLRT) Shimodaira-Hasegawa (SH)-like branch support, as implemented in PhyML 3.0 (47). SH values of 0.9 were considered statistically significant (48). To confirm mother-to-child transmission, phylogenetic analysis was done using the generated clonal *gag* and *nef* sequences from infants collected 1 month after delivery and the mother at week 32 of pregnancy or delivery or 1 month after delivery. Recombinant sequences were excluded from the analysis using the Phi test (Φ_w_) (44). The generated sequences were aligned per study participant using CLUSTAL W (45). ML phylogenetic trees were reconstructed using the best-fitting model, GTR +I +G, with GARLI v.2.01 (46). Statistical support for internal branches was determined by ML-based aLRT SH-like branch support, as implemented in PhyML 3.0 (47). SH values of 0.9 were considered statistically significant (48). HIV-1 subtyping in *gag* and *nef* was done in a previous sub-study using the population sequences obtained from the earliest infant sample (16).

Initial assessment of phylogenetic signal was done by TempEst (49). Intra-host HIV-1 population dynamics were reconstructed using Bayesian coalescent Skygrid models on individual infant sequence sets. The evolutionary rates were estimated in BEAST v.1.10.1 using the SRD06 substitution model, a relaxed uncorrelated lognormal clock model, and the Bayesian skygrid coalescent tree prior (50). For each infant sequence set, Markov chain Monte Carlo (MCMC) chains were run for 300 million steps, subsampling parameters and trees every 30,000th step. BEAGLE library v.2 was used to improve the computational time of likelihood calculations. In Tracer v.1.6, an effective sample size (ESS) of ≥100 was used to determine convergence of several evolutionary parameters. Maximum clade credibility (MCC) trees for each infant were inspected for selective sweeps across different time points. Selective sweeps in this context refer to when a given genetic variant confers a reproductive advantage within an individual, it can undergo positive selection possibly sweeping through by the quasispecies (virus population) by rising quickly in frequency generation over generation until fixation (51).

### Diversity analysis

In total, 200 mL bootstrap trees were generated for each study participant using GARLI v.2.01 (46). Diversity (substitutions per nucleotide site) was calculated in BioPerl 5.0 using pairwise patristic distances between participant-specific sequences obtained from the same sample time point (52) (the Perl script for diversity analysis is available from the authors upon request). These estimates were then summarized in R4.1.0 to obtain the mean diversity and the 95% confidence intervals.

### Transmitted/founder HIV-1 analysis

First, alignments of the infant *nef* or *gag* sequences at month 1 were made with CLUSTALW as part of Geneious Prime 2020.1.1 (https://www.geneious.com) (45). Consensus sequences were then generated for each sequence set. It was assumed that this consensus sequence was sampled before the onset of immune selection and that it corresponded to the actual *gag* or *nef* sequence of T/F virus (or viruses) responsible for establishing productive infection in the infants (17).

Second, the number of T/F viruses in either the *gag* or *nef* sequence set above were quantified using a previously published strategy (17). Briefly, alignments were visually inspected using the highlighter tool (https://www.hiv.lanl.gov/content/sequence/HIGHLIGHT/highlighter_top.html) (17). A single T/F virus was expected to evolve from the founder strain in a star-shaped phylogeny, and this was inspected using neighbor-joining trees. Furthermore, diversity estimates, i.e., pairwise Hamming distances (HD, number of base positions at which the two genomes differ), the observed maximum HD, and the overall percentage of diversity within each sample were used to classify a homogenous or single T/F variant lineage and heterogeneous viral lineage (17). Single T/F viruses were defined as a monophyletic lineage or star-shaped phylogeny with % diversity below 0.5, while multiple T/F viruses had two or more distinct lineages distinguished by three or more nucleotide polymorphisms and % diversity below 0.5. The results obtained were also confirmed by the Poisson fitter tool; here, the frequency distribution of the genetic distances between pairs of HIV-1 homogenous sequences follow an approximate Poisson distribution and star-like tree topology (17, 53).

### Estimation of the intra-host HIV evolutionary rates and selection pressure

#### Renaissance counting

Signature pattern analysis (VESPA tool) was applied to identify the amino acid positions that characterize the differences between time points among the infants, and the renaissance counting approach was then used to ascertain whether these identified positions (sites) were under positive selection. To quantify the site-specific selection pressures, i.e., the nonsynonymous and synonymous substitution rate ratios (dN/dS); a renaissance counting procedure, as implemented in BEAST V 1.10.1 (50), was used. This process maps the substitutions throughout evolutionary history and applies an empirical Bayes procedure to the counted number of nonsynonymous and synonymous substitutions (54). A dN/dS value close to 1 suggests neutral selection, a dN/dS value of significantly higher than 1 indicates positive selection, while a dN/dS value significantly lower than 1 indicates negative selection. In this analysis, the individual nucleotide substitution rates were estimated using the Hasegawa, Kishino, and Yano (HKY) substitution model with estimated base frequencies, no site rate heterogeneity, and three data partitions for the coding positions (50, 55). In addition, an uncorrelated lognormal relaxed clock model with a constant population size model as the tree prior was used. Posterior distributions were then obtained using Bayesian MCMC analysis. MCMC chains were run for 100 × 10^6^ generations and then sampled every 10,000 generations to ensure stationarity and adequate effective sample sizes >100 as diagnosed using Tracer (http://tree.bio.ed.ac.uk/software/tracer/). The uncertainty of continuous parameter estimates is expressed as 95% highest posterior density (HPD) intervals. A summary file was created by BEAST listing the mean dN/dS estimate and the credible intervals for each codon site and sites were classified as significantly negatively or positively selected when the HPD interval did not include a value of 1.

#### Hierarchical estimates of evolutionary parameters (nucleotide substitution, nonsynonymous, and synonymous rates) with and without population-specific fixed effects

Hierarchical phylogenetic models allow borrowing information from one individual to another, providing more precise within-individual level evolutionary rate estimates. The assumption in hierarchical phylogenetic modelling (HPM) is that evolutionary parameters for the infants are related through prior distributions as the infants come from the same distribution. Thus, information is pooled from other infants to obtain estimates of the mean evolutionary rate and the variation among the means for the infants included in the model. Individual evolutionary rates are therefore modeled to vary around a shared unknown but estimable population mean which results in higher precisions for the rate estimates compared to independent individual estimates (56). The mean evolutionary rate was also estimated with the HPM approach in BEAST v.1.10.1 for the HIV-1 populations within the infants. A separate strict or uncorrelated lognormal relaxed clock model and a constant population size model as the tree prior were assigned to the sequence set containing all available sequences from an individual. The parameters of the molecular clock model and the HKY substitution model and codon substitution models were then created hierarchically across lineages, with all other parameters varying independently across each infant.

To further disentangle the molecular adaptation process, the substitution rate of every branch in a tree can be divided into expected nonsynonymous (E[N]) and synonymous (E[S]) substitution rates, reflecting the respective contribution of E[N] and E[S] substitution rates to the overall substitution rate. Although comparisons between E[N] and E[S] rate estimates can be difficult to interpret since they are uncorrected for the number of possible nonsynonymous and synonymous alterations, relative differences between, e.g., patient groups can still be explored. We determined E[N] and E[S] rate estimates as described by Lemey et al. (28).

#### Patient classification for HPM analysis with fixed effects

Within the BEAST platform, one can incorporate a mixed-effects HPM to estimate and compare the evolutionary rate, synonymous, or nonsynonymous substitution rate (response variable) between patient population groups (defined by different predictor variables) (27, 57). The HPM evolutionary rate on the log scale (response variable) was treated as a continuous variable and CD4% decline (slower vs faster) and presence or absence of CTL escape variants (yes vs no) were defined as binary predictor or indicator variables. A data-driven approach was used to group the infants into slower or faster progressors based on the CD4% decline rate (CD4% dynamics over time). We used a complete linkage hierarchical k-shape clustering algorithm based on the shape-based distances from the predicted CD4% values using the dtwclust v.5.5.6 R package (58). Predictions were done using the generalized additive models (GAM) with integrated smoothness estimation assumption of linearity between predictor and response variable using the mgcv v.1.8-35 R package (Fig. S8B).

### CTL escape mutation analysis

CTL escape mutations were defined as polymorphisms that lead to resistance to immune recognition which are selected by CTLs, and defined as known escape variants within epitopes as documented in the CTL/CD8+ epitope variant and escape mutation list at: http://www.hiv.lanl.gov/content/immunology/variants/ctl_variant.html (last updated at 25 March 2021, 23:53:17–06). The presence of potential CTL (CD8+ T cell) escape variants in both infant and mother HIV-1 Gag and Nef sequences was determined by pairwise comparison of the HLA restricted epitope sequences and patient sequences from all time points. For each HLA restricted epitope, we summarized the type of variant if any, the frequency of the variant in the infant, the time point at which the variant is detected, and results from the interferon-gamma response ELISPOT, which was conducted with wild-type peptides representing subtype A and D viruses. Several amino acid differences (CTL variants) between infant, maternal and HXB2 sequences were detected (data available on request).

### Statistical analysis

Statistical analyses were performed in R v4.1.0 using the two-tailed Mann-Whitney U-test or Wilcoxon rank-sum test for comparing means of a continuous outcome variable between two independent groups. Kruskal Wallis H test was used to compare the means of a continuous outcome variable between three or more independent groups. GAM with integrated smoothness estimation in the mgcv R package was used to classify participants into slow or fast progressors based on their CD4% dynamics over time. GAM adapts the fitted curve to the data and relaxes the assumption of linearity between predictor and response variable. Participants with less than three CD4% measurement were excluded because of insufficient unique time values to support the cubic smoothing (knots, k = 3). Hierarchical clustering on predicted values from GAM were used for the classification.

## Data Availability

The newly generated sequences are available in GenBank, accession numbers PP742038 to PP744431. For confidentiality reasons and to protect the identity of the study participants, clinical and demographic data beyond those published in this article are not publicly available. However, additional data can be made available for peers upon request and mutual confidentiality agreement.

## SUPPLEMENTAL MATERIAL

Figures S1 to S5 jvi.00072-24-s0001.docx Clones, viral load, neighbor-joining trees, diversity, and phylogenetic trees.

Figure S6 jvi.00072-24-s0002.docx Selective sweeps in gag and nef over time.

Figures S7 to S9 jvi.00072-24-s0003.docx Host evolutionary rate and substitution rates.

Supplemental tables jvi.00072-24-s0004.docx Tables S1 to S6.

ASM does not own the copyrights to Supplemental Material that may be linked to, or accessed through, an article. The authors have granted ASM a non-exclusive, world-wide license to publish the Supplemental Material files. Please contact the corresponding author directly for reuse.
