# Supplementary material for: Higher HIV-1 evolutionary rate is associated with cytotoxic T lymphocyte escape mutations in infants
Source: J Virol. 2024 May 30;98(7):e00072-24. doi: 10.1128/jvi.00072-24 (PMC11265422; doi:10.1128/jvi.00072-24)
Supplement: Figures S1 to S5 — Clones, viral load, neighbor-joining trees, diversity, and phylogenetic trees. [file jvi.00072-24-s0001.docx]

**HIGHER HIV-1 EVOLUTIONARY RATE IS ASSOCIATED WITH CYTOTOXIC T LYMPHOCYTE ESCAPE MUTATIONS IN INFANTS**

**Authors:** Jamirah Nazziwa (1), Sophie M. Andrews (2), Mimi M. Hou (2), Christian A. W. Bruhn (1), Miguel A. Garcia-Knight (2,3), Jennifer Slyker (4,11), Sarah Hill (5), Barbara Lohman Payne (6,7), Dorothy Mbori-Ngacha (6), Philippe Lemey (8), Grace John-Stewart (4,7,9,10,11), Sarah L. Rowland-Jones (2)*, and Joakim Esbjörnsson (1, 2)§*

*Authors with equal contribution

**Author Affiliations:** (1) Department of Translational Medicine, Lund University, Sweden; (2) Nuffield Department of Clinical Medicine, University of Oxford, UK; (3) Department of Microbiology and Immunology, University of California San Francisco, California, USA; (4) Department of Global Health, University of Washington, Seattle, Washington, United States of America; (5) Department of Pathobiology and Population Sciences, Royal Veterinary College, UK; (6) Department of Paediatrics and Child Health, University of Nairobi, Nairobi, Kenya; (7) Department of Medicine, University of Washington, Seattle, Washington, United States of America; (8) Department of Microbiology, Immunology and Transplantation, Rega Institute, KU Leuven, Leuven, Belgium; (9) Department of Pediatrics, University of Washington, Seattle, Washington, USA; (10) Global Center for Integrated Health of Women, Adolescents and Children (Global WACh), University of Washington, Seattle, Washington, USA; (11) Department of Epidemiology, University of Washington, Seattle, Washington, USA.

**§Corresponding Author:**

Joakim Esbjörnsson

BMC B13

Department of Translational Medicine

Lund University

221 84 Lund, Sweden

Email: [Joakim.esbjornsson@med.lu.se](mailto:Joakim.esbjornsson@med.lu.se)

**SUPPLEMENTARY FIGURES 1-5**

**Figure S1A. Number of *gag* and *nef* clones successfully collected from each infant per time-point.** Tile plot showing the number of clones collected per time-point for each infant. Grey coloured tiles represent the time-points where the clonal sequences are missing because no plasma was collected or that plasma was depleted. Grey tiles marked with X indicate time-points where PCR and sequencing failed. The tiles are coloured by time-point in both infant and mother as indicated on the y-axis. Abbreviations: p32, 32 weeks of pregnancy; del, delivery; pn1, one month after delivery.


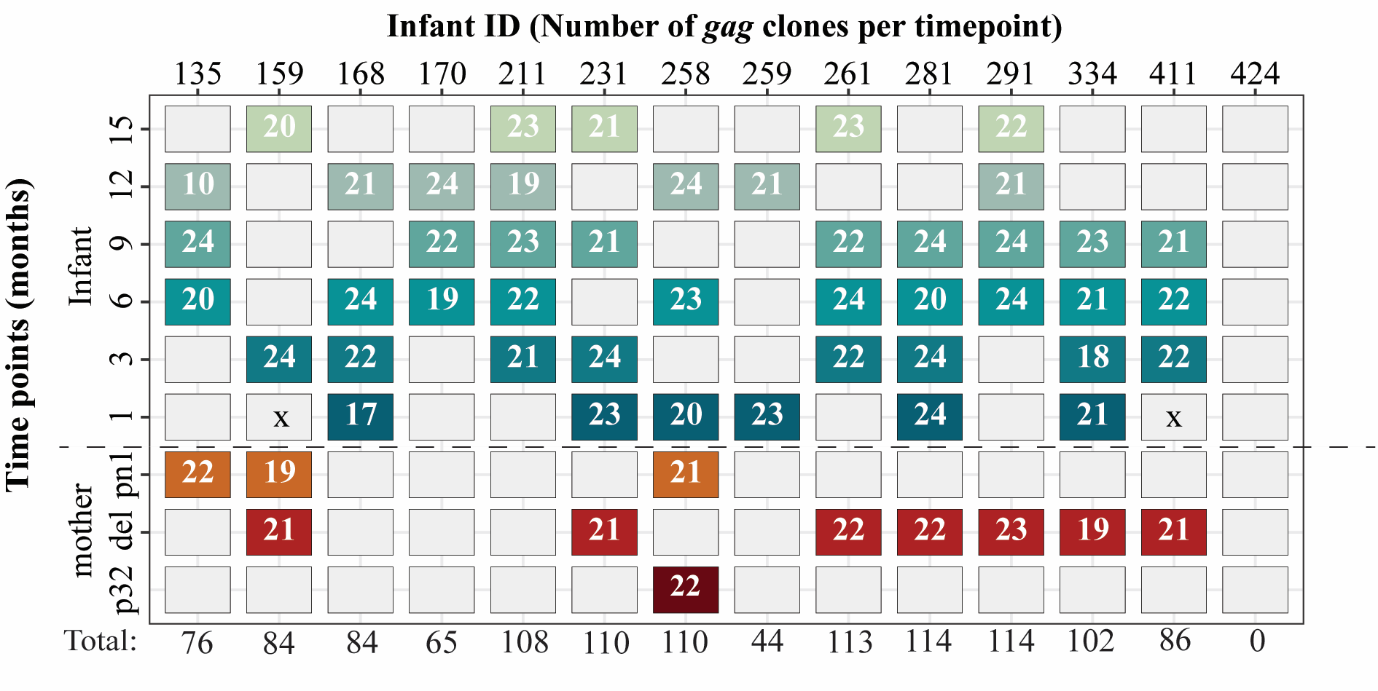


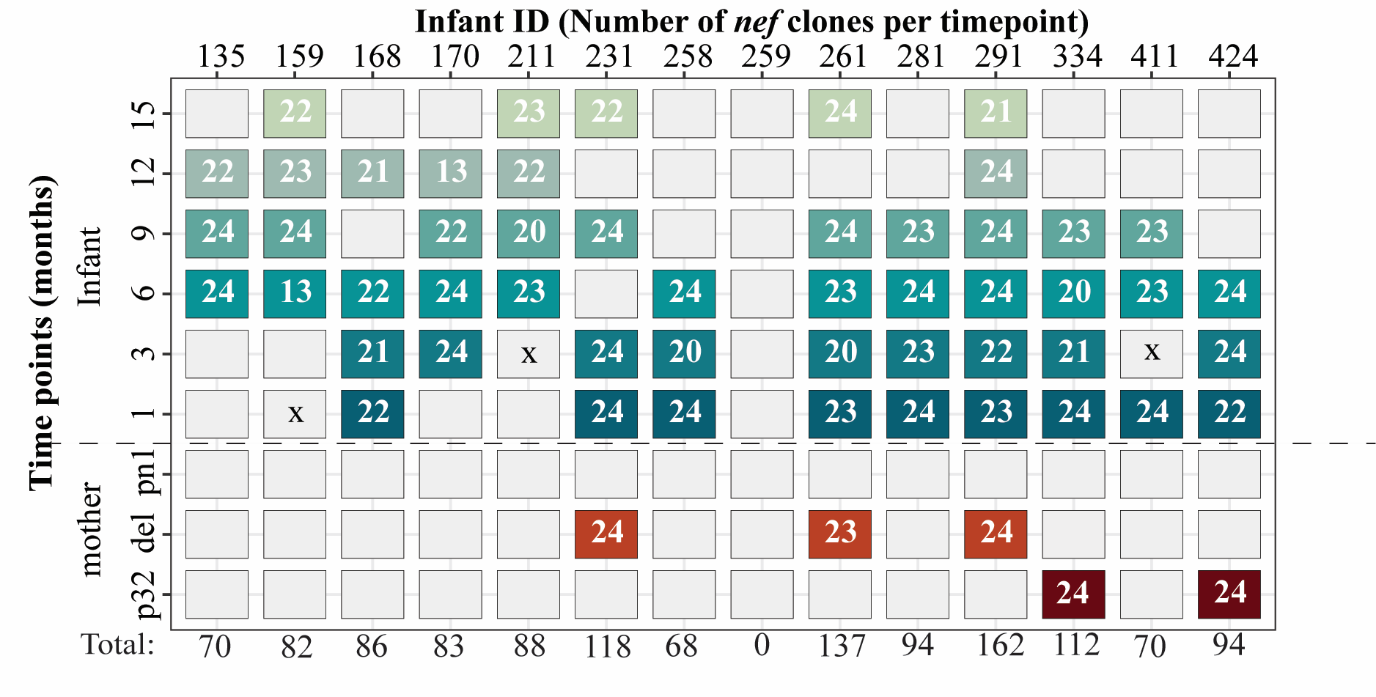


**Figure S1B. Maternal viral load measured before and after delivery.** Plot showing maternal viral load measurements taken during and after pregnancy.

**
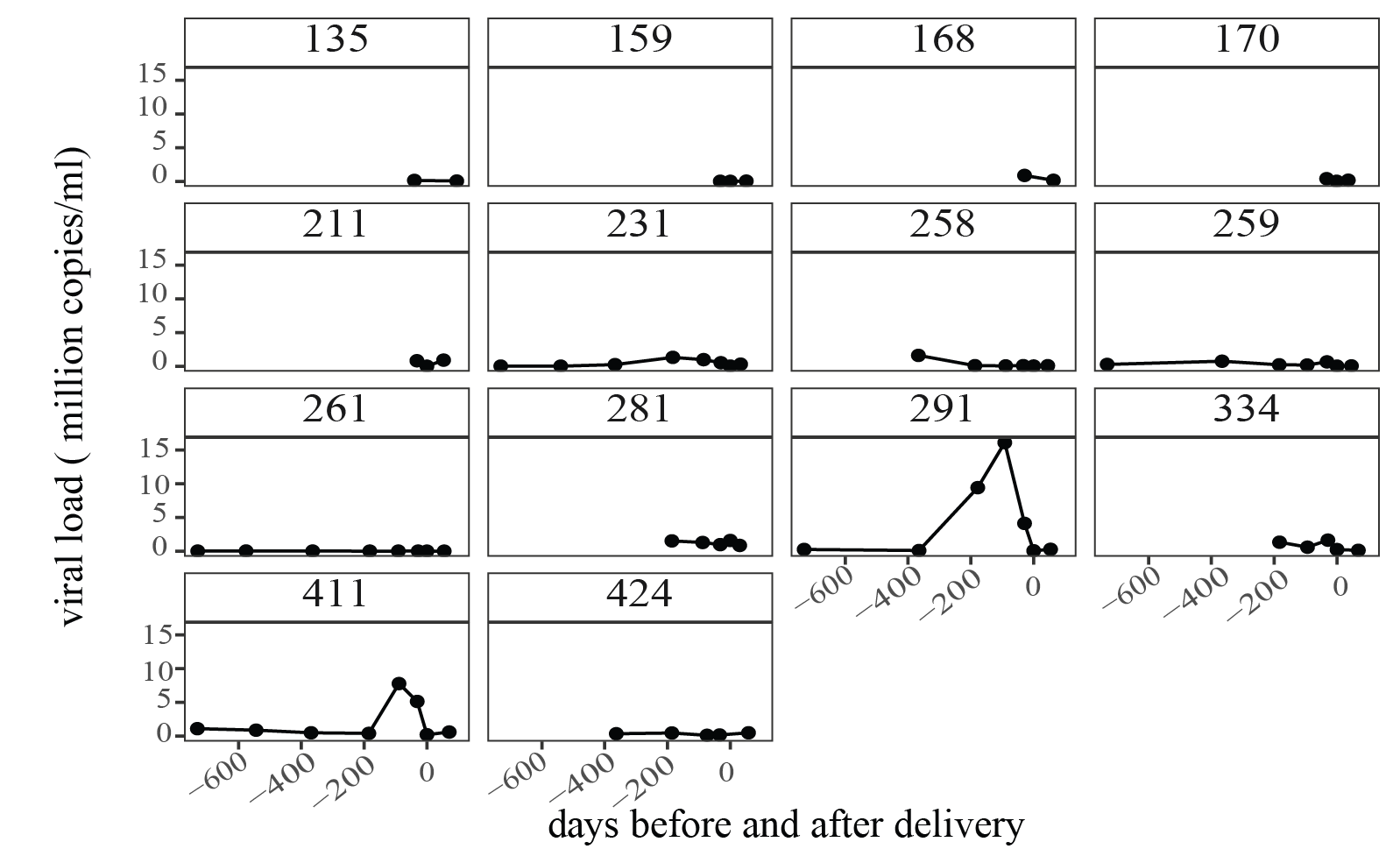
**

**Figure S2A. Neighbour-joining trees and Highlighter analyses plot for the *gag* sequences for five infants infected by single viruses**. Infant 334 first sample at month 1 after birth; infant 168 first sample at month 1 after birth; infant 231 first sample at week 2 after birth; infant 259 first sample at month 1 after birth; infant 281 first sample at month 1 after birth. This analysis enables one to find the nucleotides in infant sequences that do not match with those in the consensus (the first sequence). The nucleotides that do not match with the consensus are assigned a colour (red=T, green=A, blue=C, yellow=G).

**
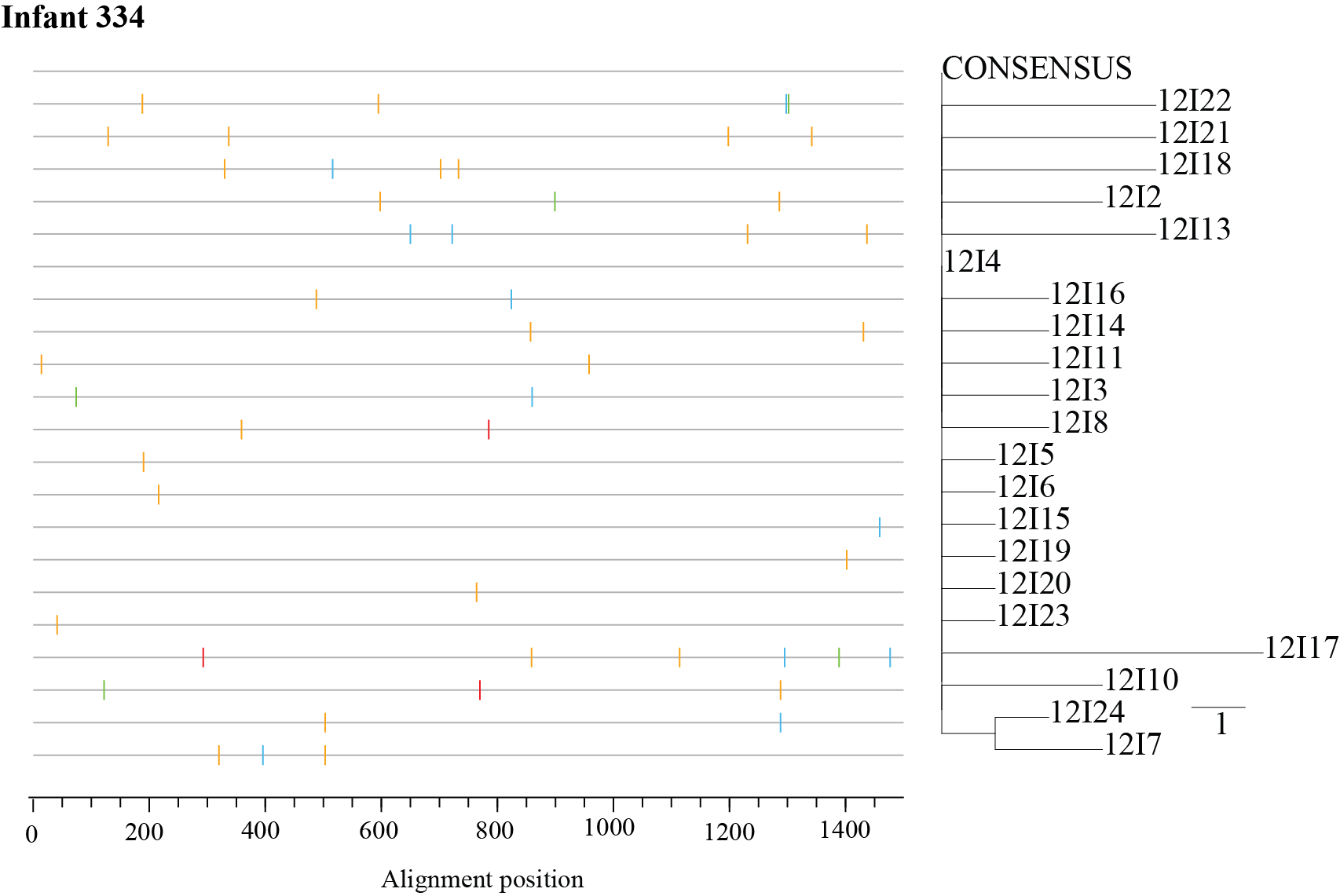
**


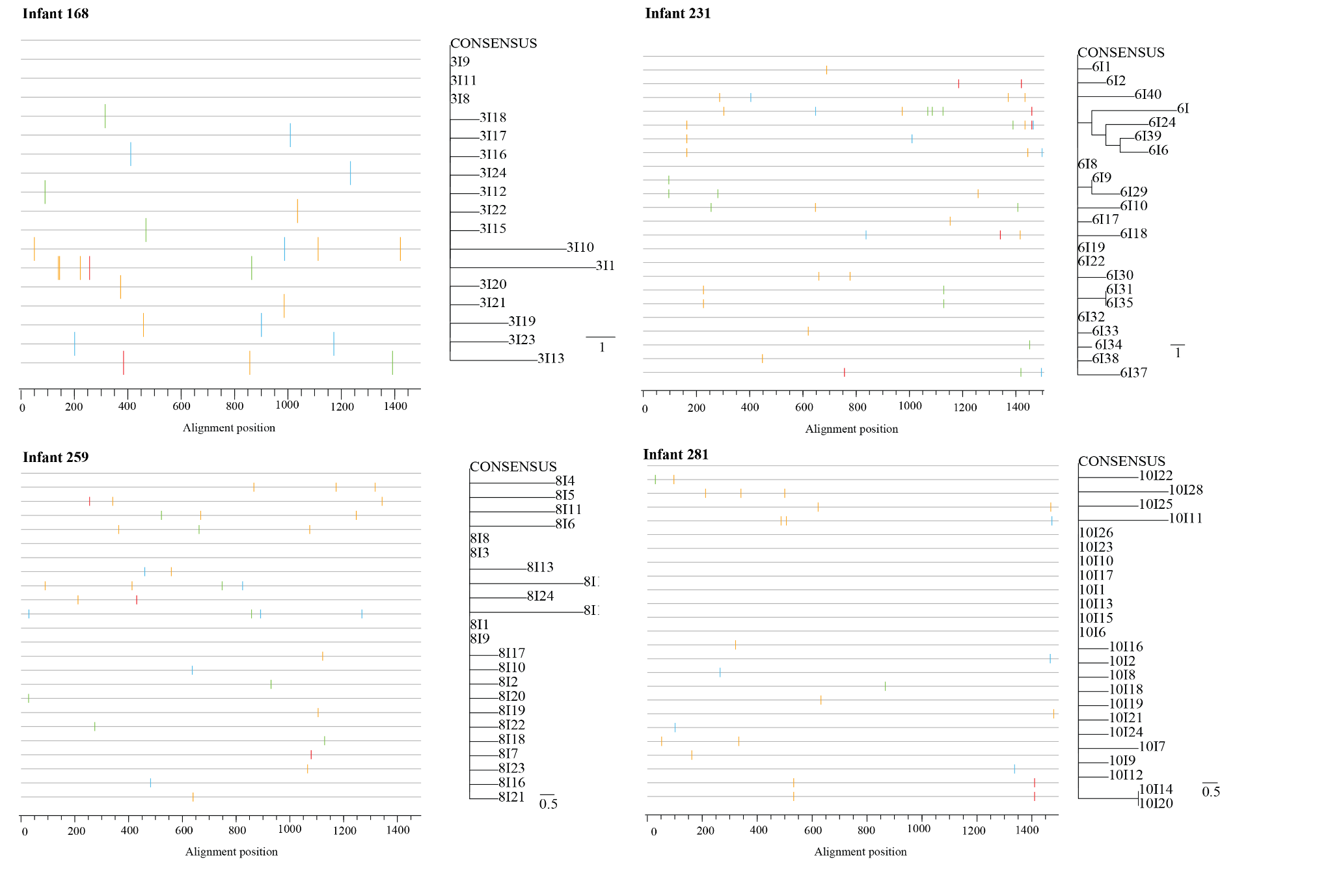


**Figure S2B. Neighbour-joining trees and Highlighter analyses plot for the *gag* sequences for one infant infected by multiple viruses**. Infant 258 first sample at month 1 after birth with infection by three viruses with recombination in sequence 7I22. The nucleotides that do not match with the consensus are assigned a color (red=T, green=A, blue=C, yellow=G).


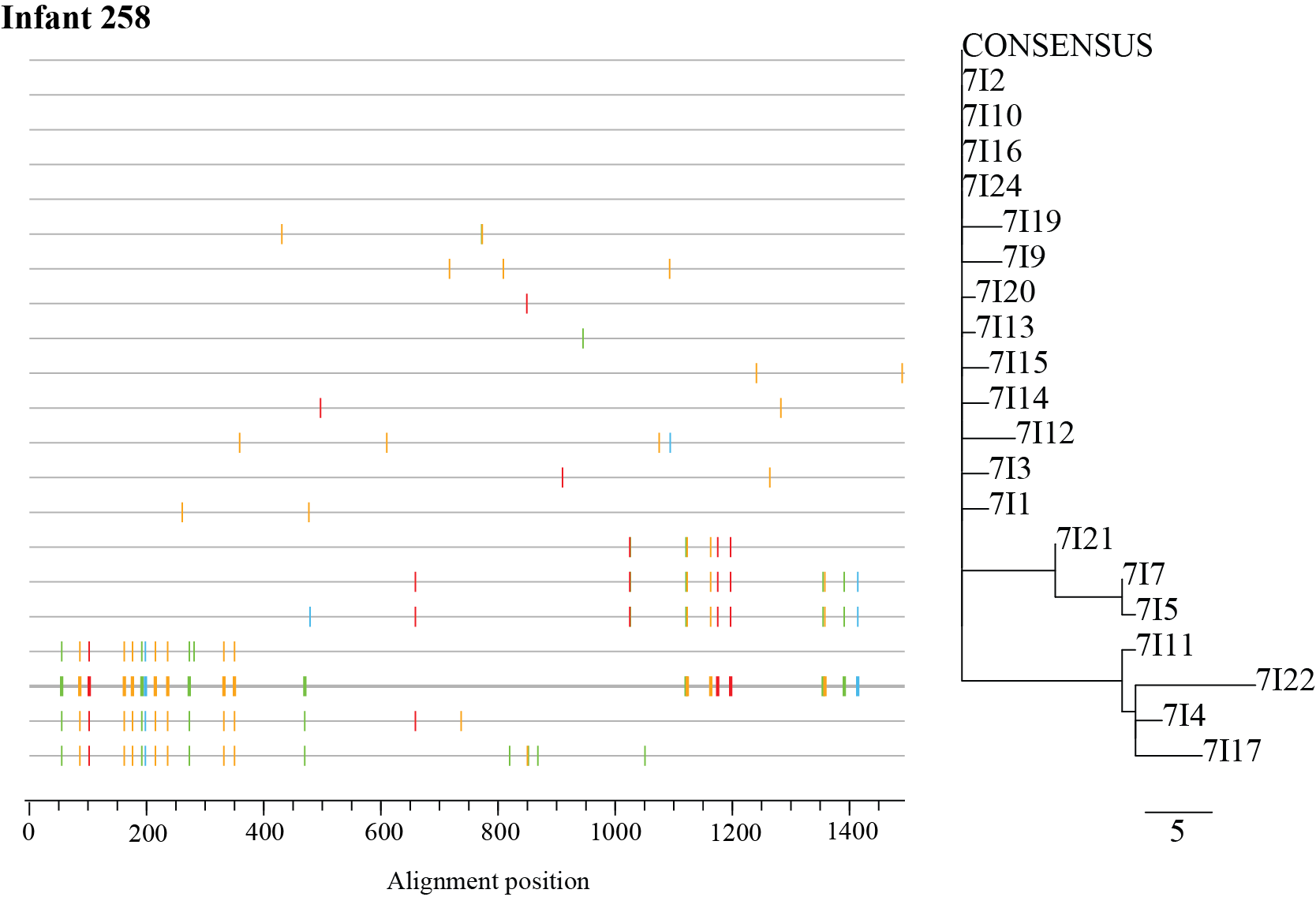


**Figure S2C. Neighbour-joining trees and Highlighter analyses plot for the *nef* sequences for seven infants infected by single viruses**. Infant 168 first sample at month 1 after birth; infant 261 first sample at month 1 after birth; Infant 281 first sample at month 1 after birth; infant 291 first sample at month 1 after birth; infant 334 first sample at month 1 after birth; infant 411 first sample at month 1 after birth; infant 424 first sample at month 1 after birth. This analysis enables one to find the nucleotides in infant sequences that do not match with those in the consensus (the first sequence). The nucleotides that do not match with the consensus are assigned a color (red=T, green=A, blue=C, yellow=G).


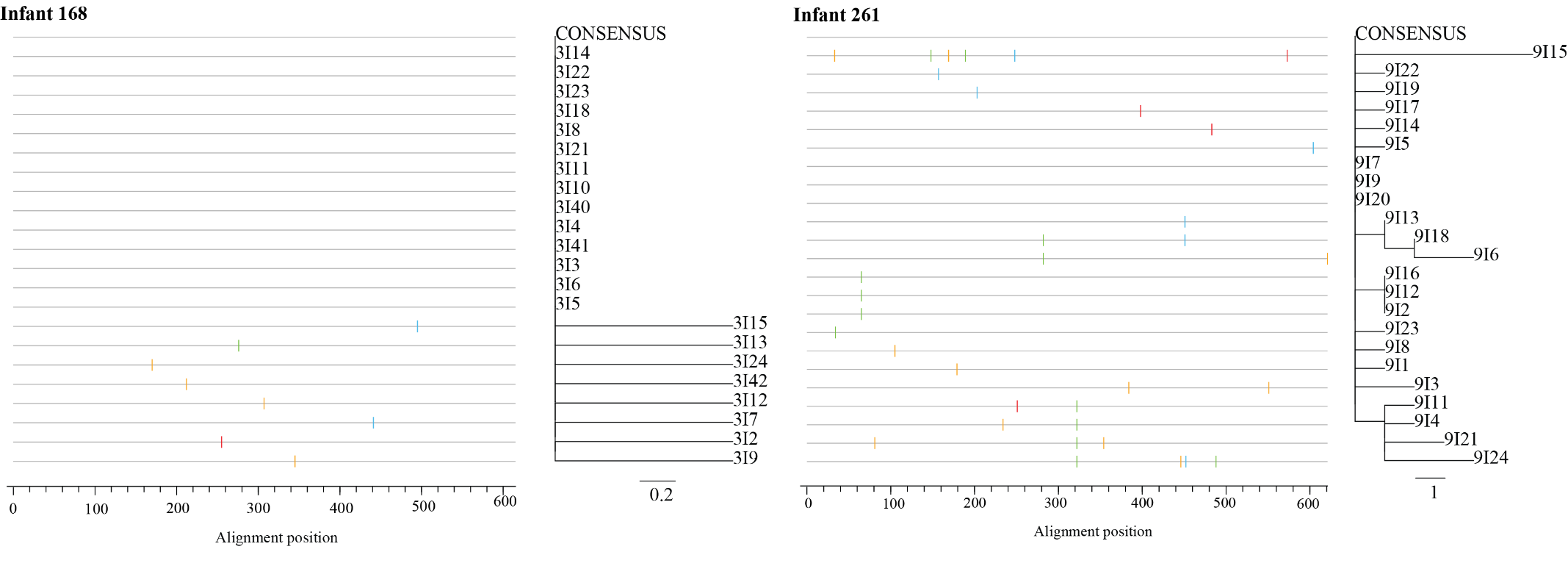


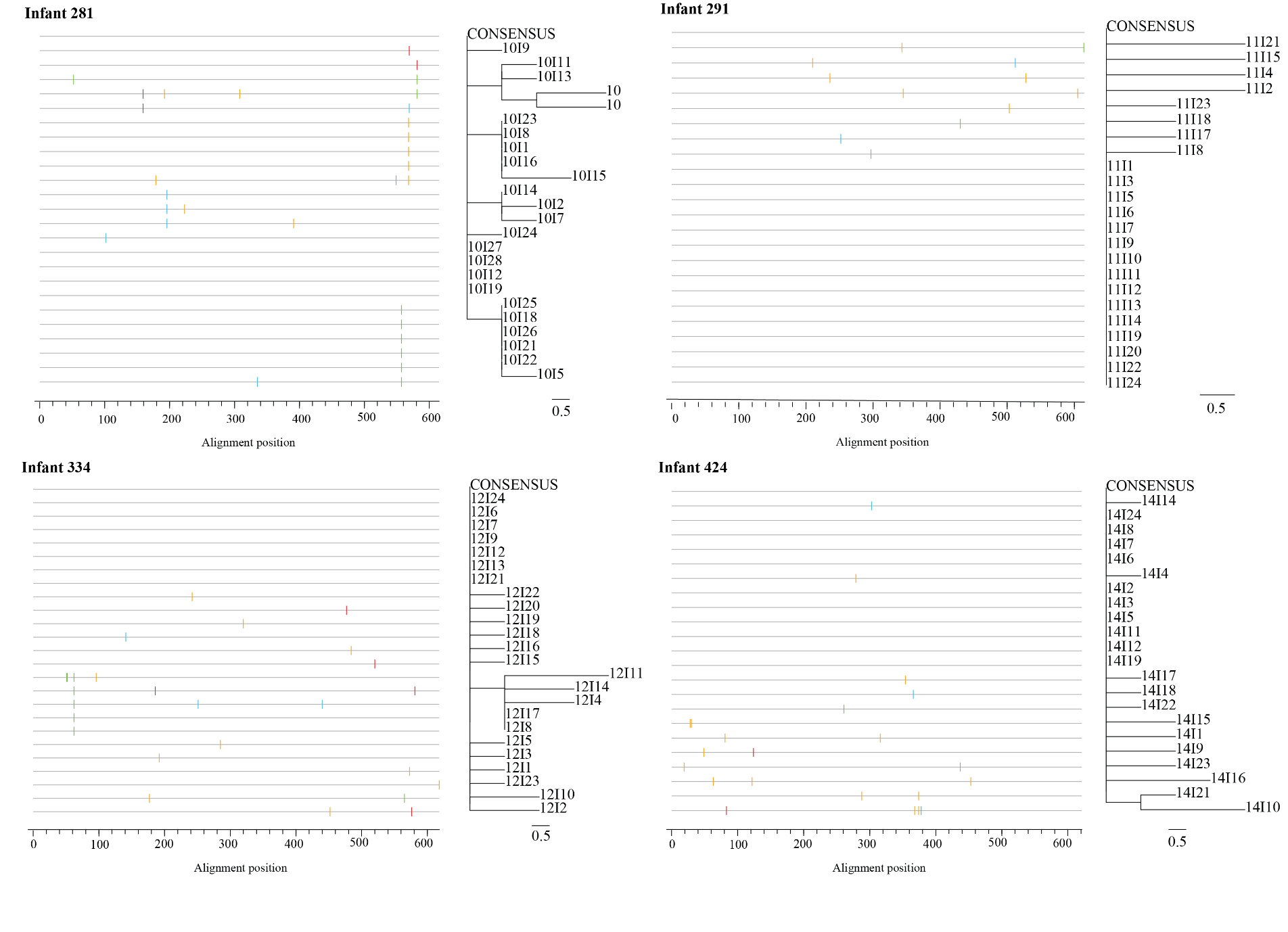


**Figure S2D. Neighbour-joining trees and Highlighter analyses plot for the *nef* sequences from two infants infected by multiple viruses**. Infant 231 first sample collected at week 2 with infection by three viruses and evidence of recombination in sequence 6I14. Infant 258 first sample at month 1 after birth with infection by three viruses. This analysis enables one to find the nucleotides in infant sequences that do not match with those in the consensus (the first sequence). The nucleotides that do not match with the consensus are assigned a color (red=T, green=A, blue=C, yellow=G).

**
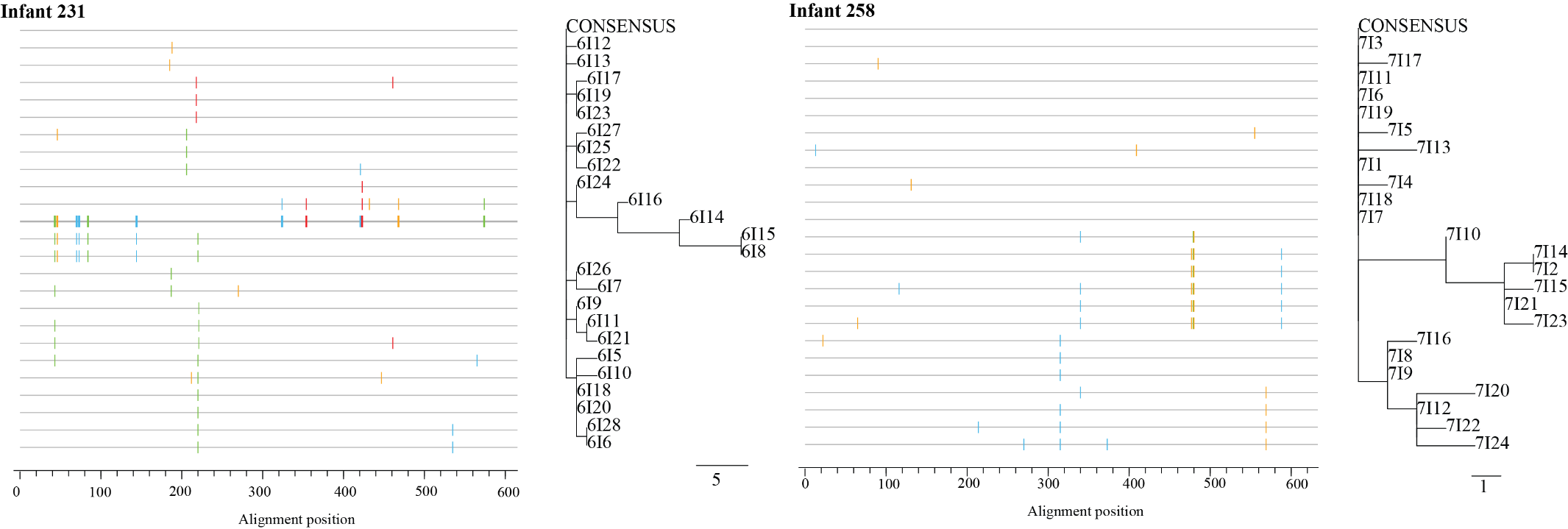
**

**Figure S3. Mean *gag* and *nef* diversity over time.** This combined box plot illustrates the sequence diversity over time in months for two distinct genetic regions, gag (depicted in blue) and nef (depicted in red). The x-axis represents the time in months, while the y-axis represents the mean sequence diversity in substitutions per site. Diversity was measured in each infant per time-point in 10-15 clones.


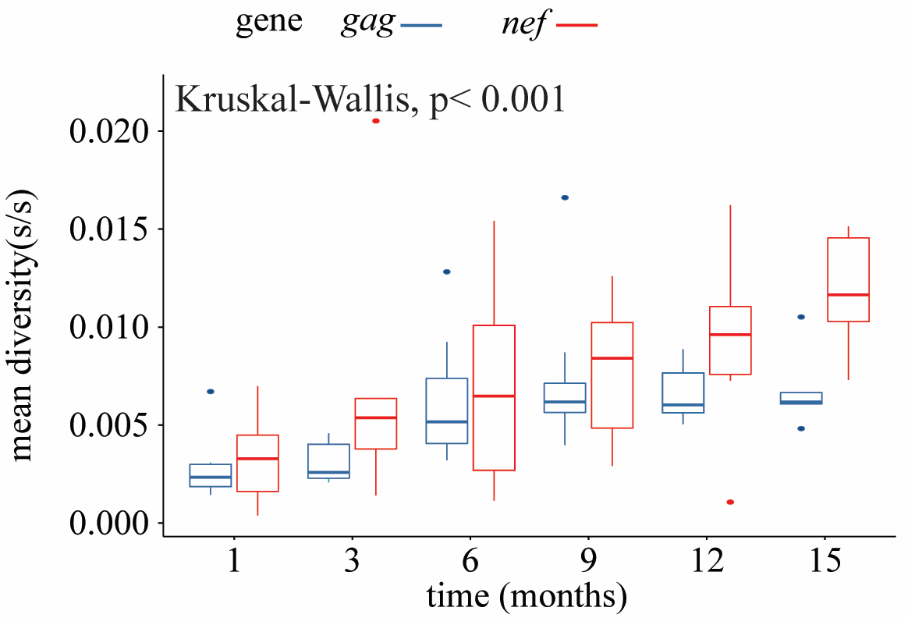


**Figure S4. Infant mean *gag* and *nef* diversity.** Pirate plots showing the distribution of *gag* (blue) and *nef* (red) diversity estimates for each infant (indicated as black dots in each plot). The horizontal line in each pirate plot indicates the mean diversity estimate.


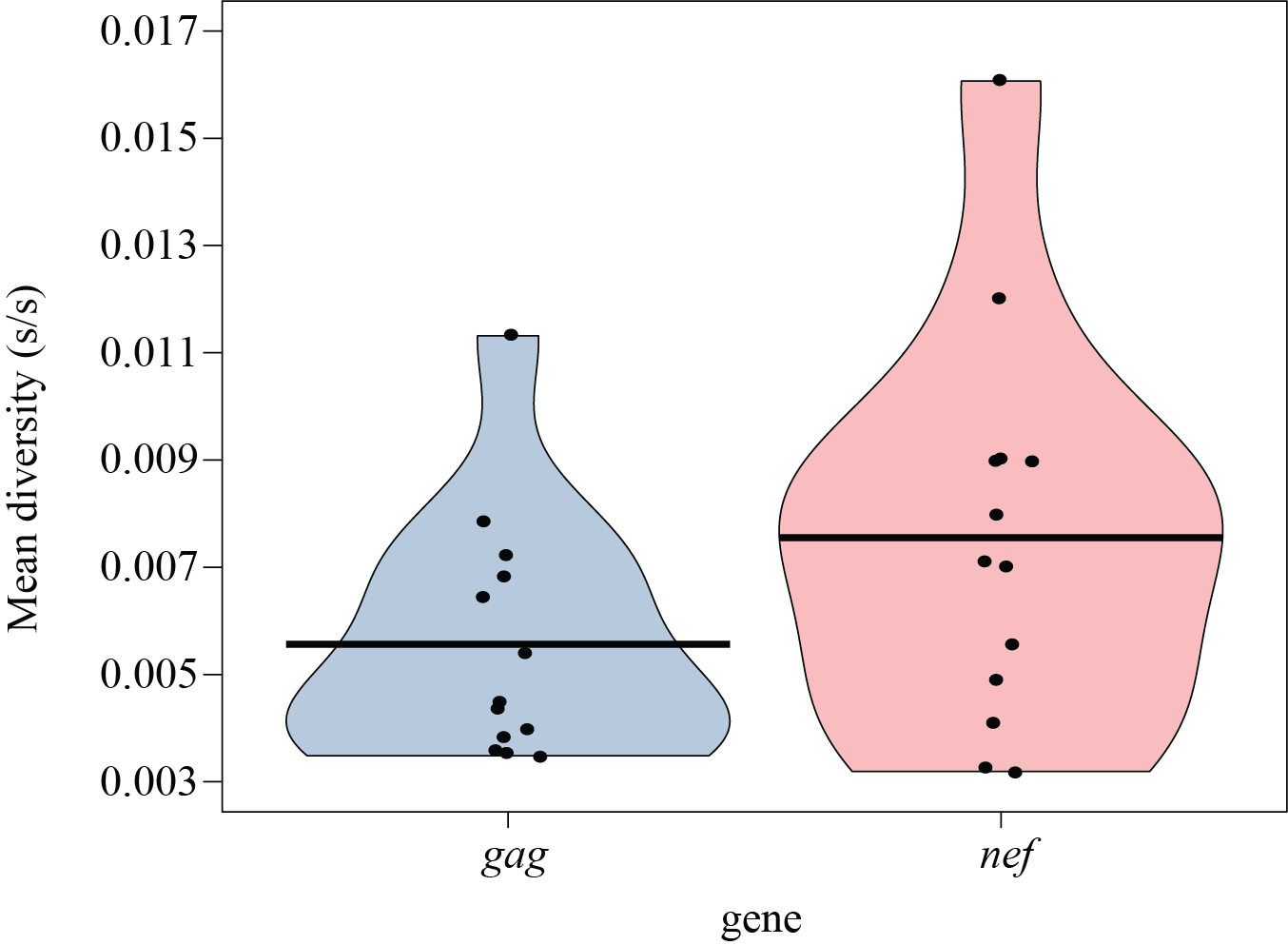


**Figure S5.** **Phylogenetic tree topology classes between mother and infant transmission pairs.** ML phylogenetic trees for two paired mother and infant sequences exemplifying the two different tree topologies observed. The red tips represent mother sequences whereas the green tips represent sequences of the paired infant. In the paraphyletic-polyphyletic (PP) class, the infant sequences were nested in more than one cluster among the mother sequences (A). In the paraphyletic-monophyletic (PM) class, the infant sequences were nested in one cluster among the mother sequences (B). Relevant branches supported by an approximate likelihood ratio test (aLRT-SH) score of 1 are indicated by “1” in the trees. The scale bar represents substitutions per site. Abbreviations: p32, 32 weeks of pregnancy; del, delivery; pn1, one month after delivery.

**
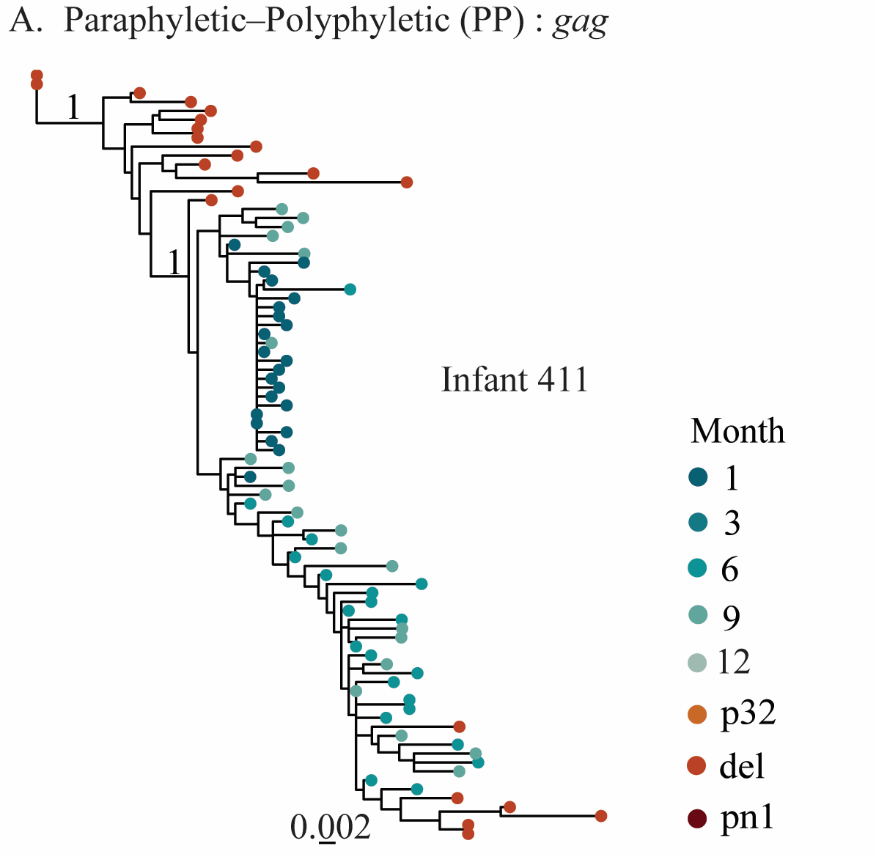
**

**
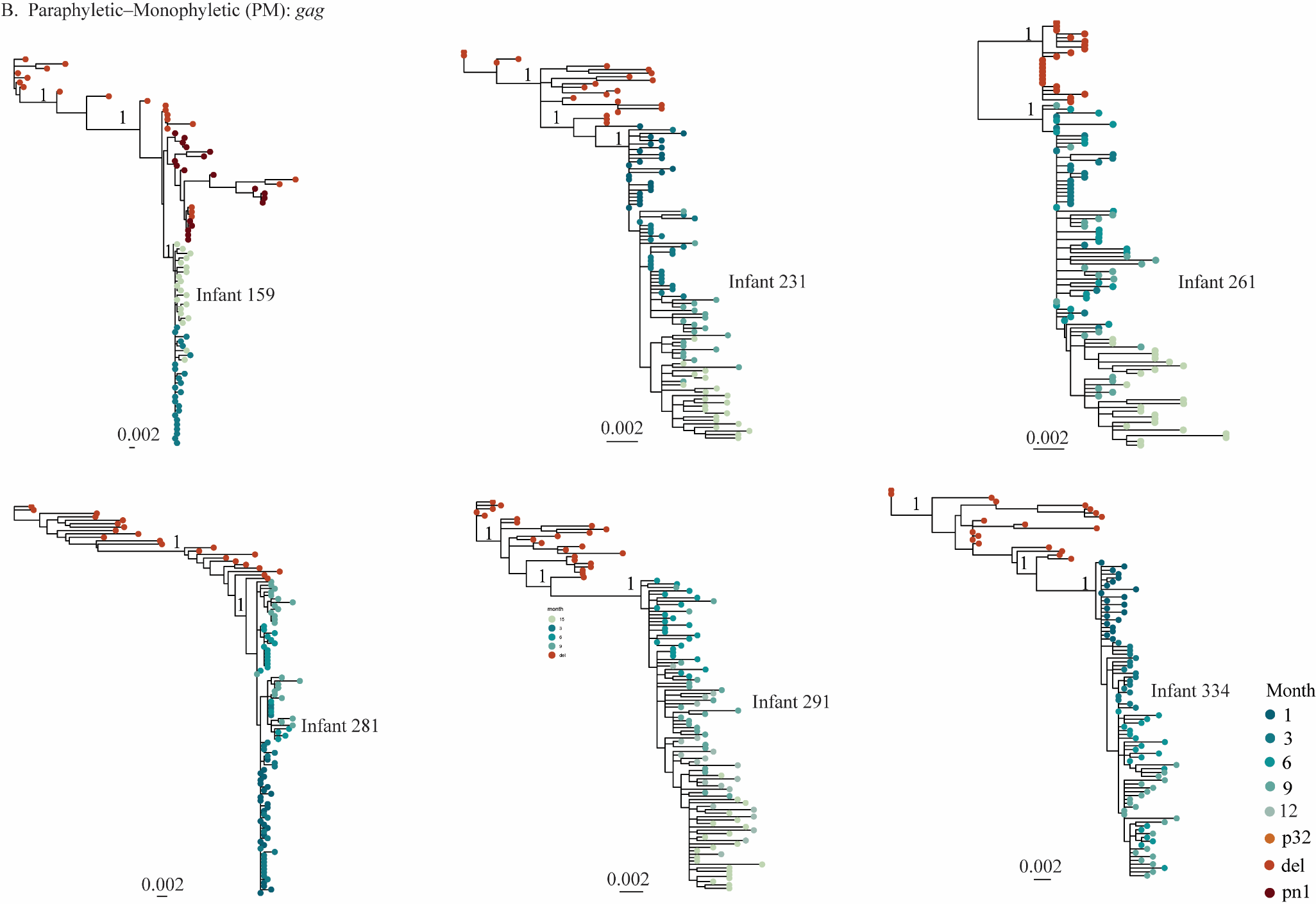
**

**
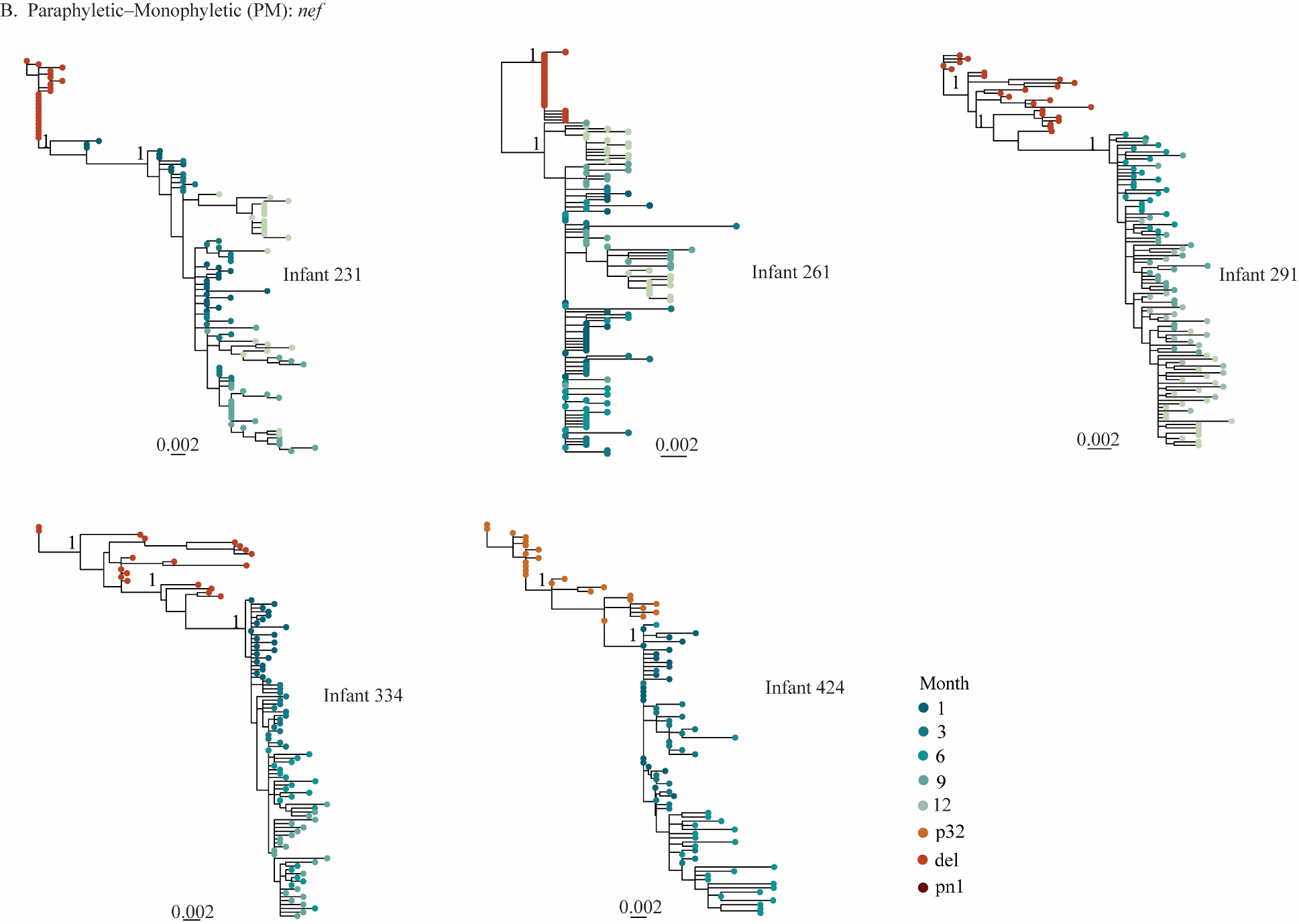
**
