# Supplementary material for: Higher HIV-1 evolutionary rate is associated with cytotoxic T lymphocyte escape mutations in infants
Source: J Virol. 2024 May 30;98(7):e00072-24. doi: 10.1128/jvi.00072-24 (PMC11265422; doi:10.1128/jvi.00072-24)

**HIGHER HIV-1 EVOLUTIONARY RATE IS ASSOCIATED WITH CYTOTOXIC T LYMPHOCYTE ESCAPE MUTATIONS IN INFANTS**

**Authors:** Jamirah Nazziwa (1), Sophie M. Andrews (2), Mimi M. Hou (2), Christian A. W. Bruhn (1), Miguel A. Garcia-Knight (2,3), Jennifer Slyker (4,11), Sarah Hill (5), Barbara Lohman Payne (6,7), Dorothy Mbori-Ngacha (6), Philippe Lemey (8), Grace John-Stewart (4,7,9,10,11), Sarah L. Rowland-Jones (2)*, and Joakim Esbjörnsson (1, 2)§*

*Authors with equal contribution

**Author Affiliations:** (1) Department of Translational Medicine, Lund University, Sweden; (2) Nuffield Department of Clinical Medicine, University of Oxford, UK; (3) Department of Microbiology and Immunology, University of California San Francisco, California, USA; (4) Department of Global Health, University of Washington, Seattle, Washington, United States of America; (5) Department of Pathobiology and Population Sciences, Royal Veterinary College, UK; (6) Department of Paediatrics and Child Health, University of Nairobi, Nairobi, Kenya; (7) Department of Medicine, University of Washington, Seattle, Washington, United States of America; (8) Department of Microbiology, Immunology and Transplantation, Rega Institute, KU Leuven, Leuven, Belgium; (9) Department of Pediatrics, University of Washington, Seattle, Washington, USA; (10) Global Center for Integrated Health of Women, Adolescents and Children (Global WACh), University of Washington, Seattle, Washington, USA; (11) Department of Epidemiology, University of Washington, Seattle, Washington, USA.

**§Corresponding Author:**

Joakim Esbjörnsson

BMC B13

Department of Translational Medicine

Lund University

221 84 Lund, Sweden

Email: [Joakim.esbjornsson@med.lu.se](mailto:Joakim.esbjornsson@med.lu.se)

**SUPPLEMENTARY FIGURE 6**

**Figure S6A. Selective sweeps in *gag* over time.** Intra-patient intra-host HIV-1 *gag* evolutionary dynamics in all infants. The line graph indicates the number of effective HIV-1 with the highest posterior density (HPD) intervals on a log scale over time. The maximum clade credibility (MCC) phylogenetic tree is layered over the graph to indicate how the virus evolved within the patient over time. The insert on the left shows amino acid sites under positive and negative selection in *gag.* The blue lines indicate sites under positive selection; grey lines are for sites under negative selection; red line show sites under neutral selection.

**
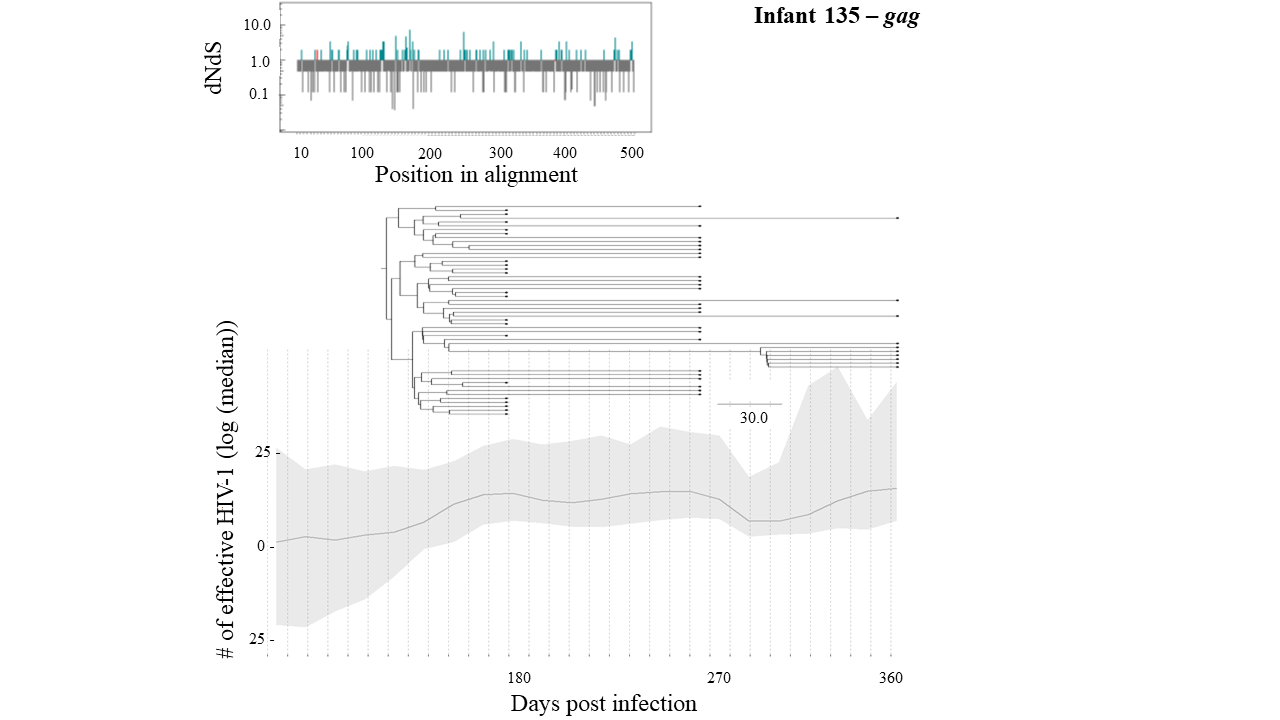

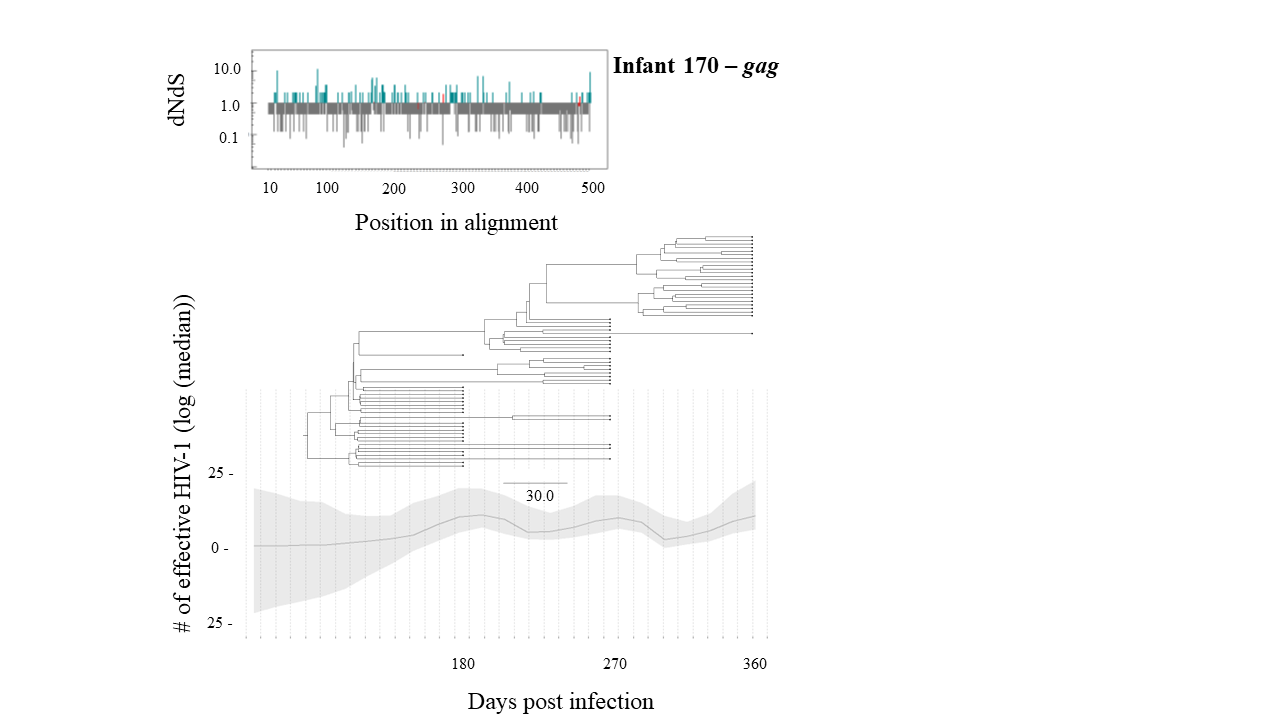
**


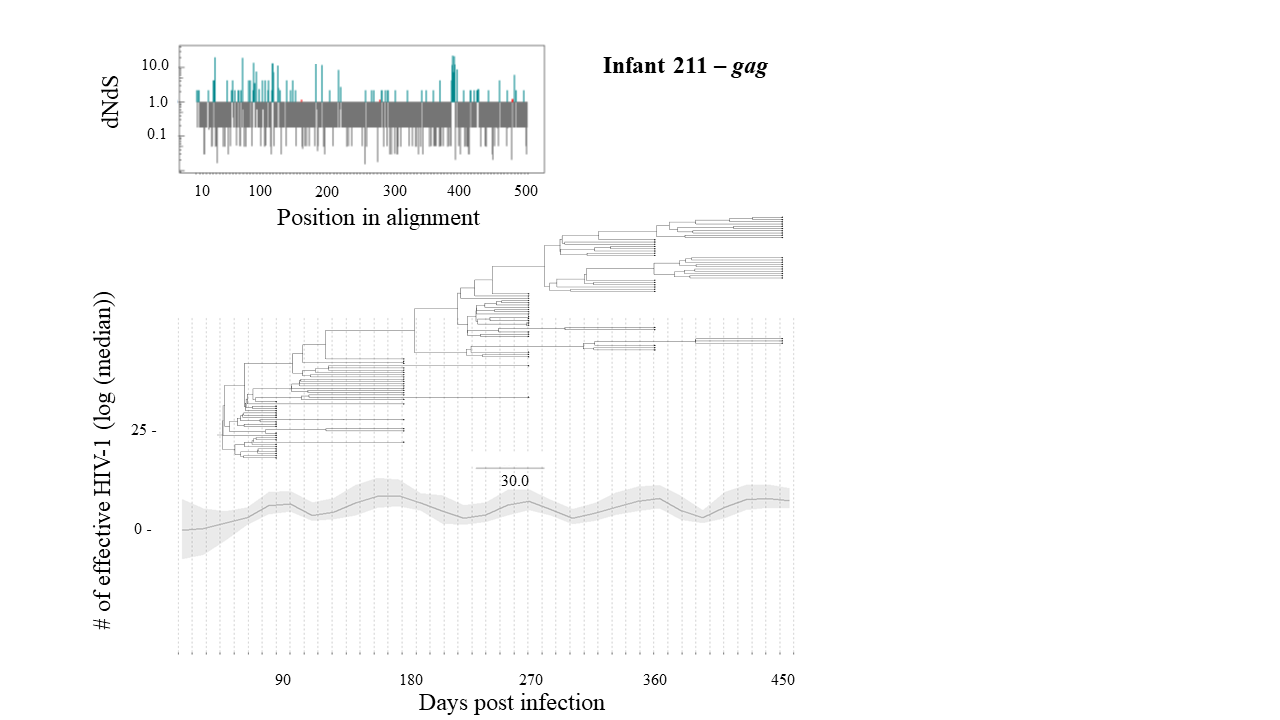

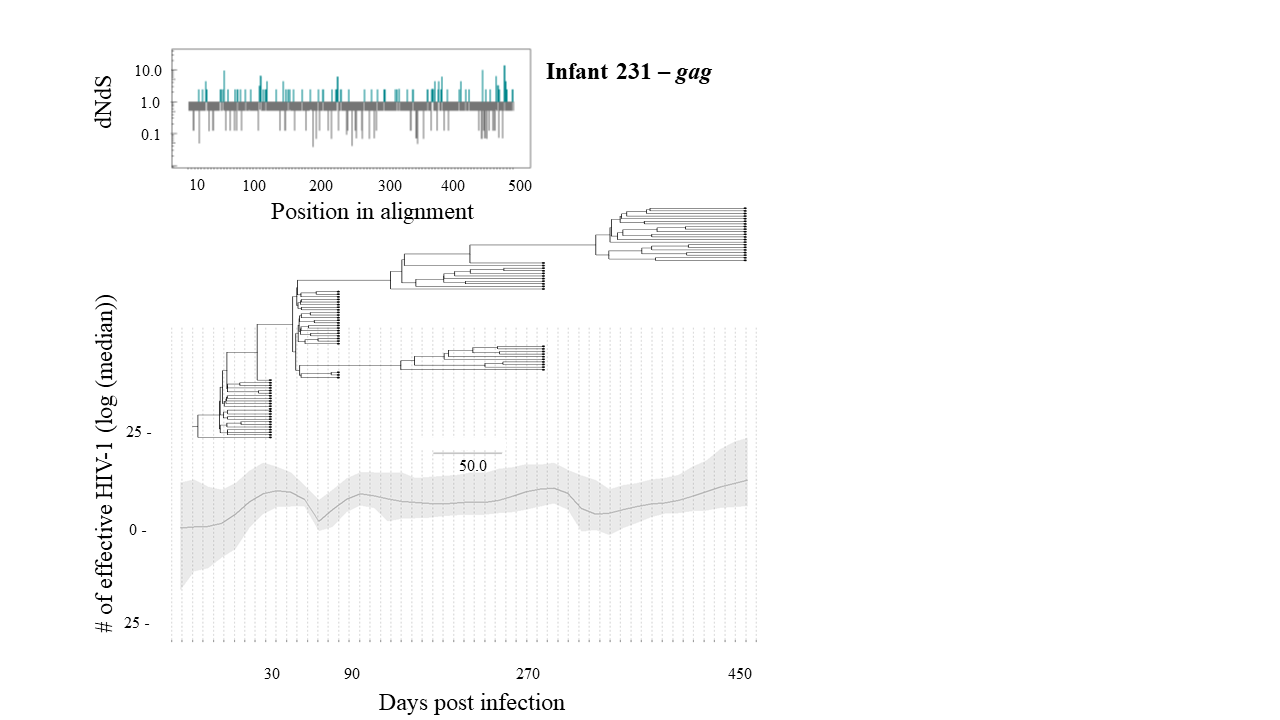


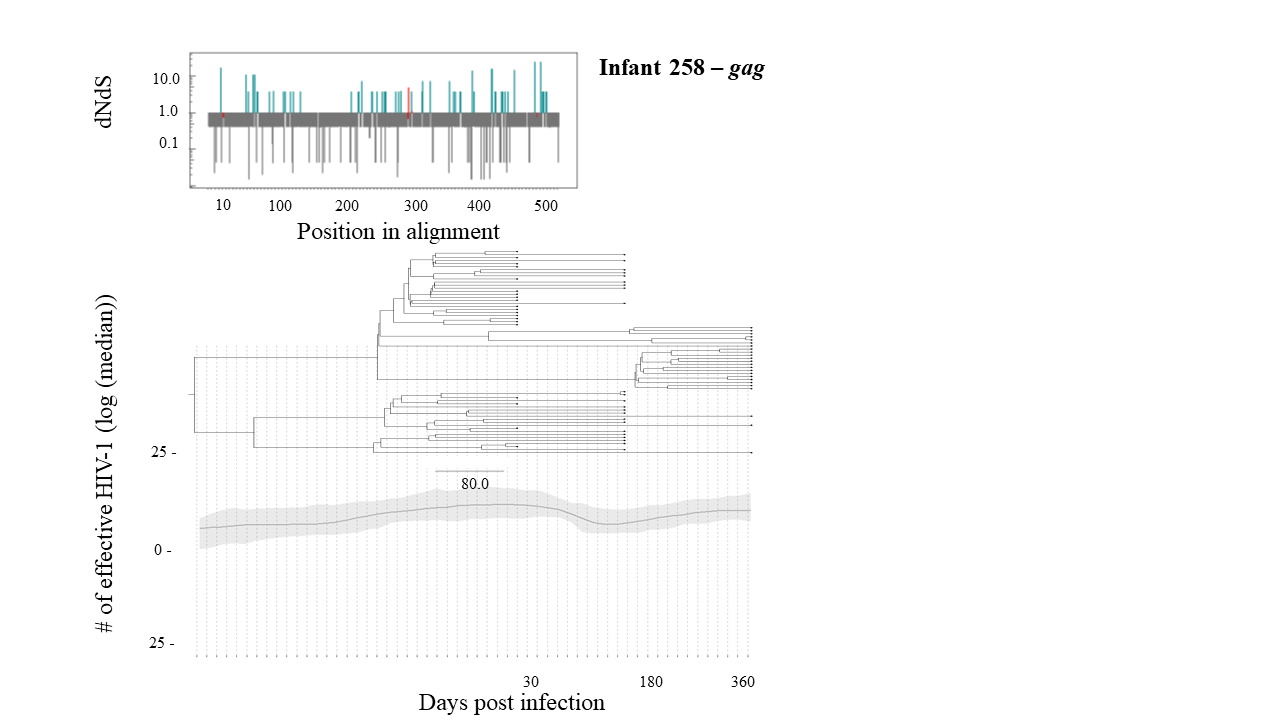

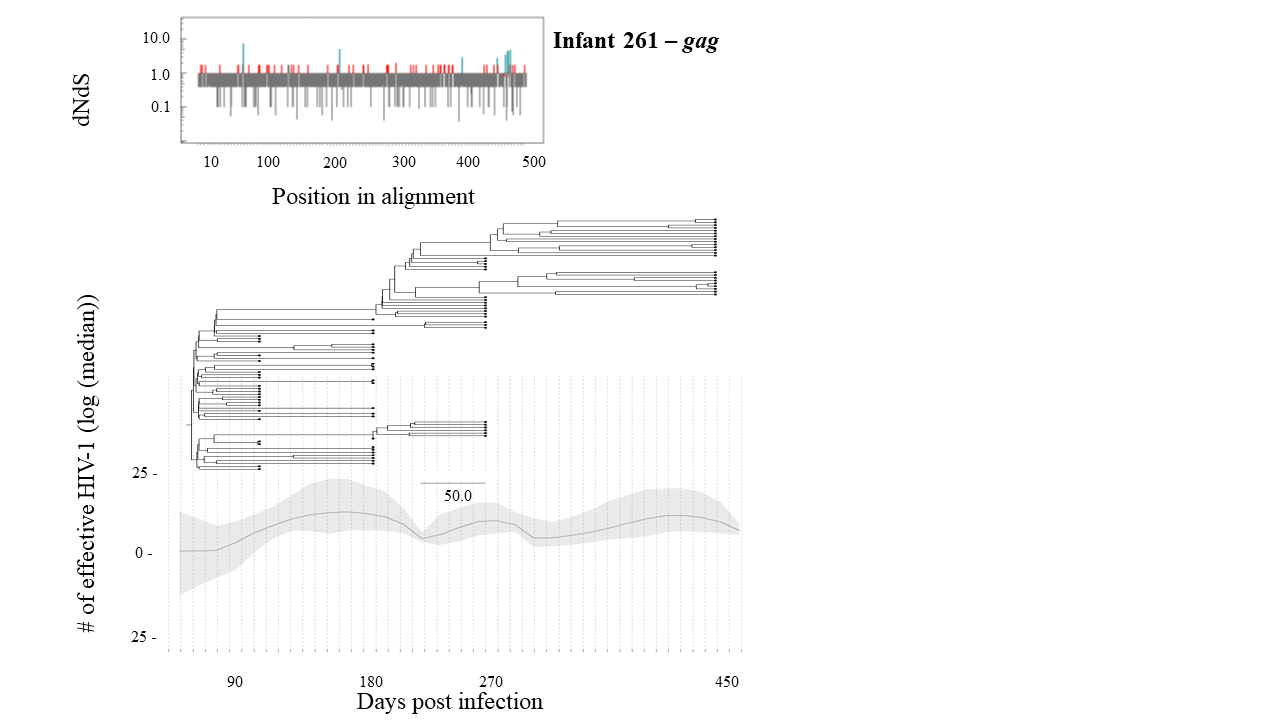


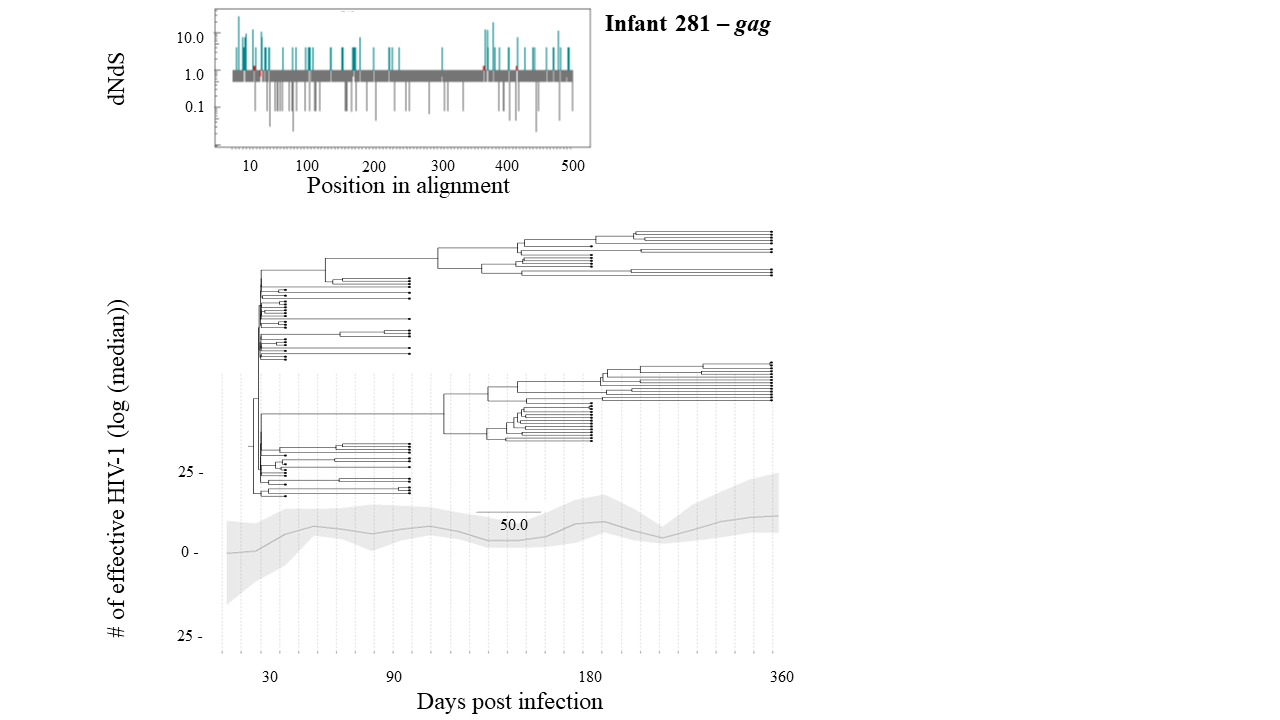

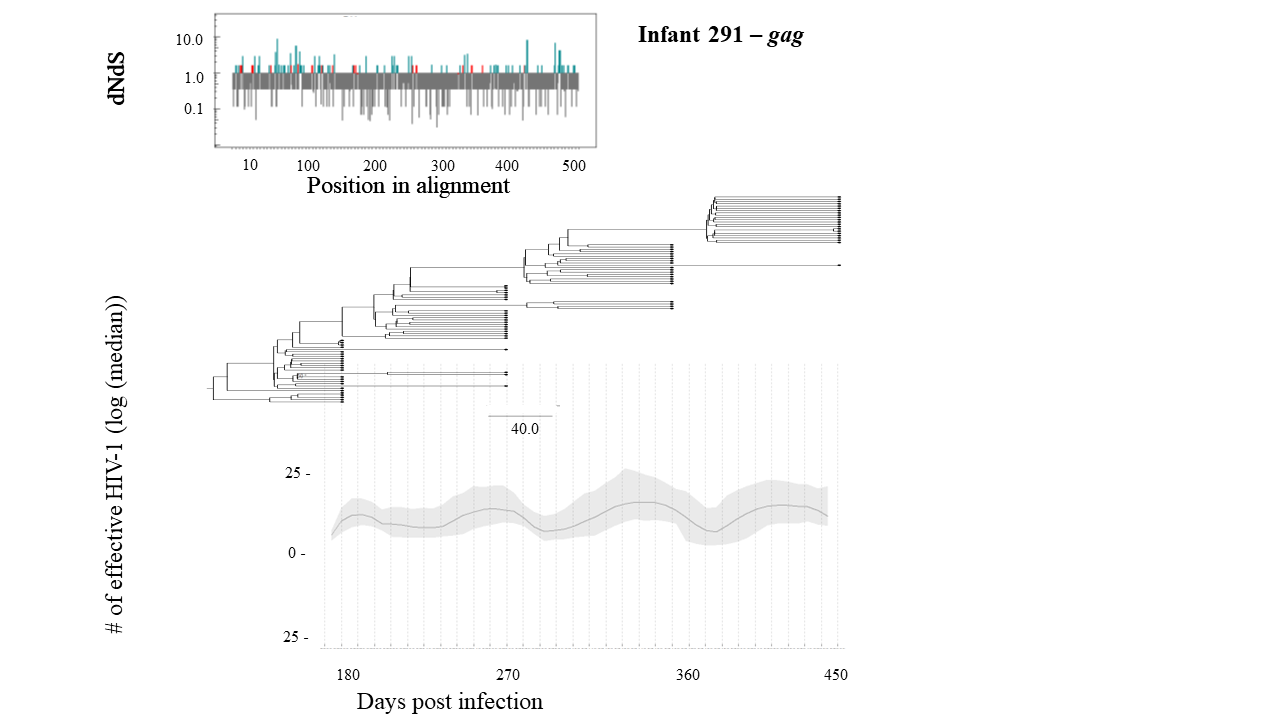


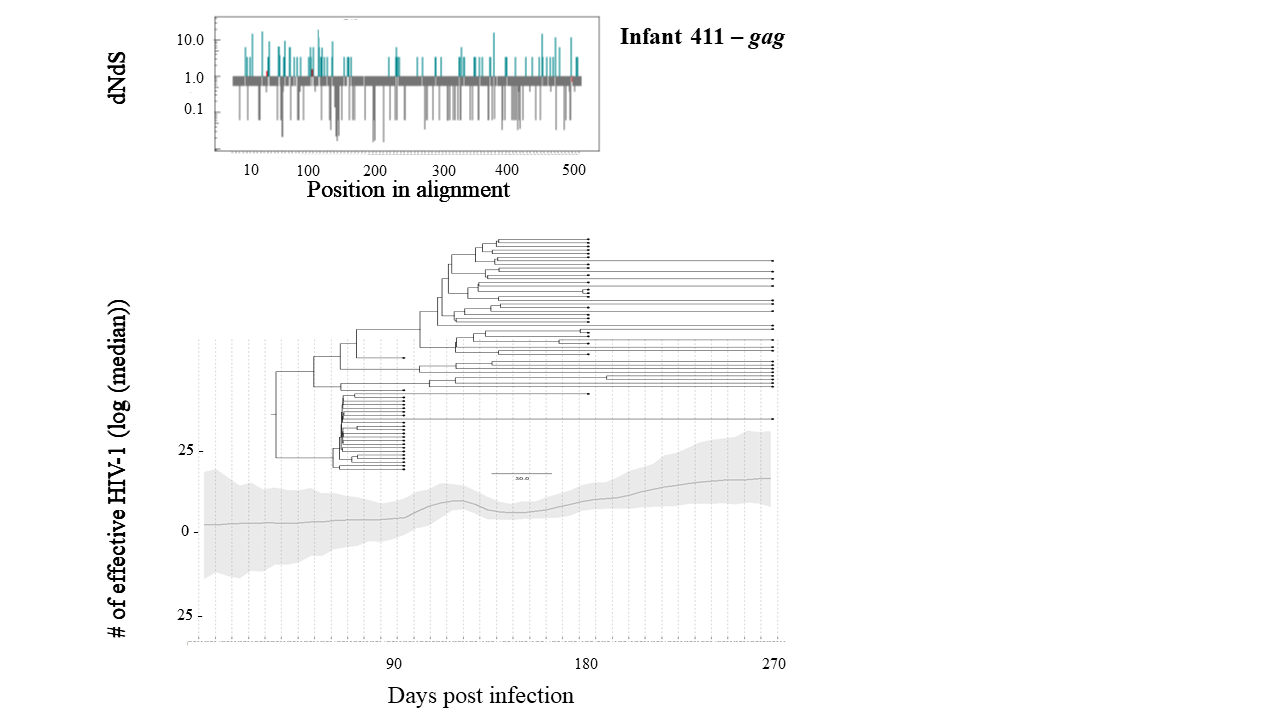


**Figure S6B. Selective sweeps in *nef* over time, and intra-patient intra-host HIV-1 *nef* evolutionary dynamics in all infants**. The line graph indicates the number of effective HIV-1 with HPD intervals on a log scale over time. An MCC phylogenetic is layered over the graph to indicate how the virus evolved within the patient over time. The insert on the left shows, amino acid sites under positive and negative selection in *nef.* The blue lines, sites under positive selection; grey lines, sites under negative selection; red line, sites under neutral selection.


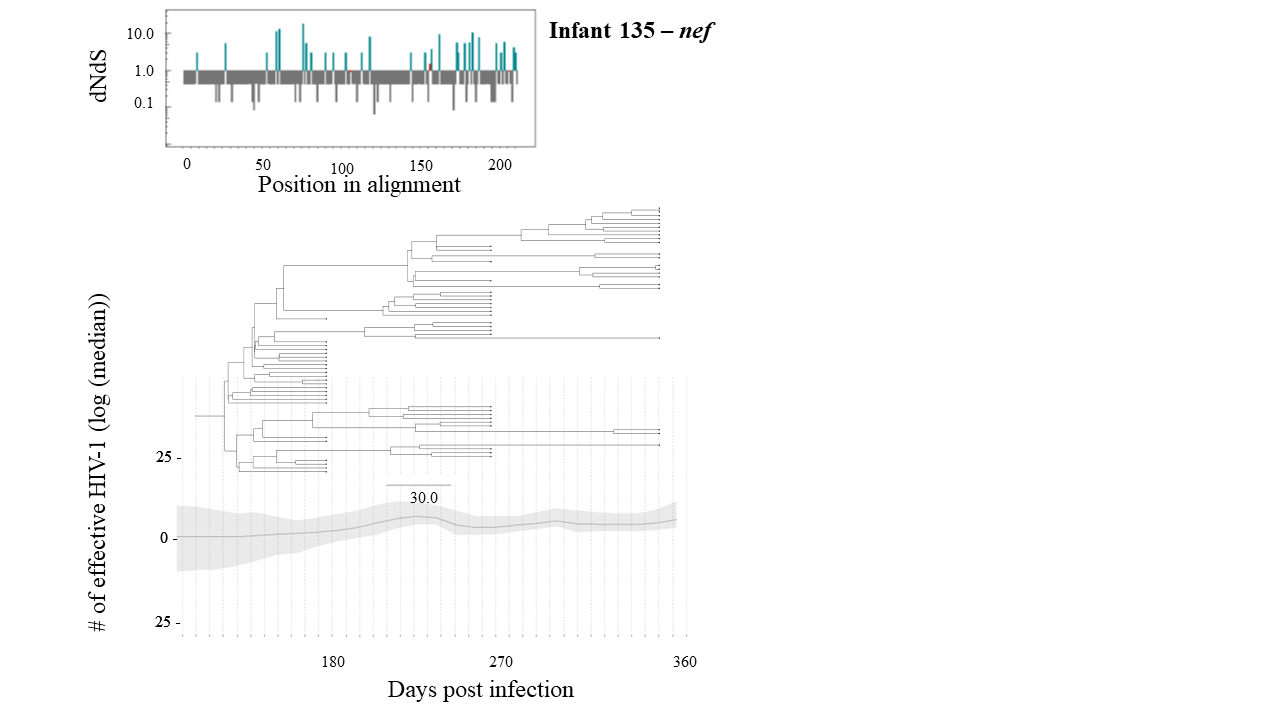

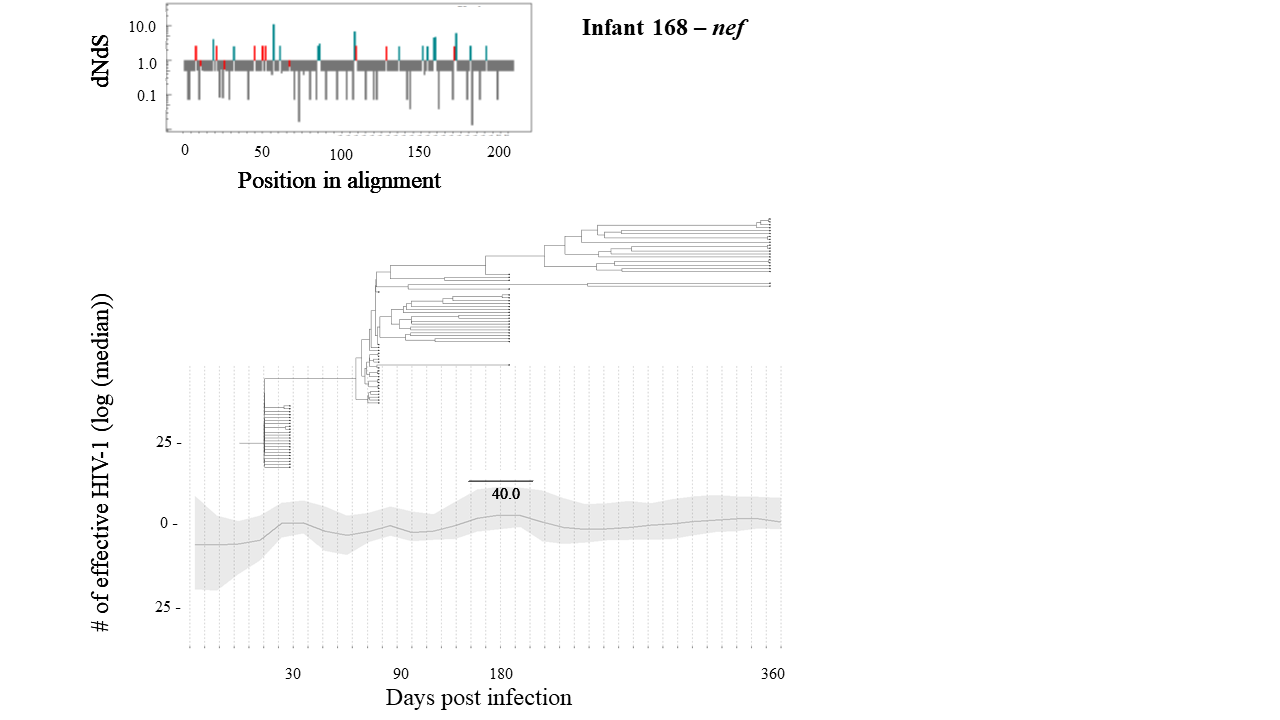


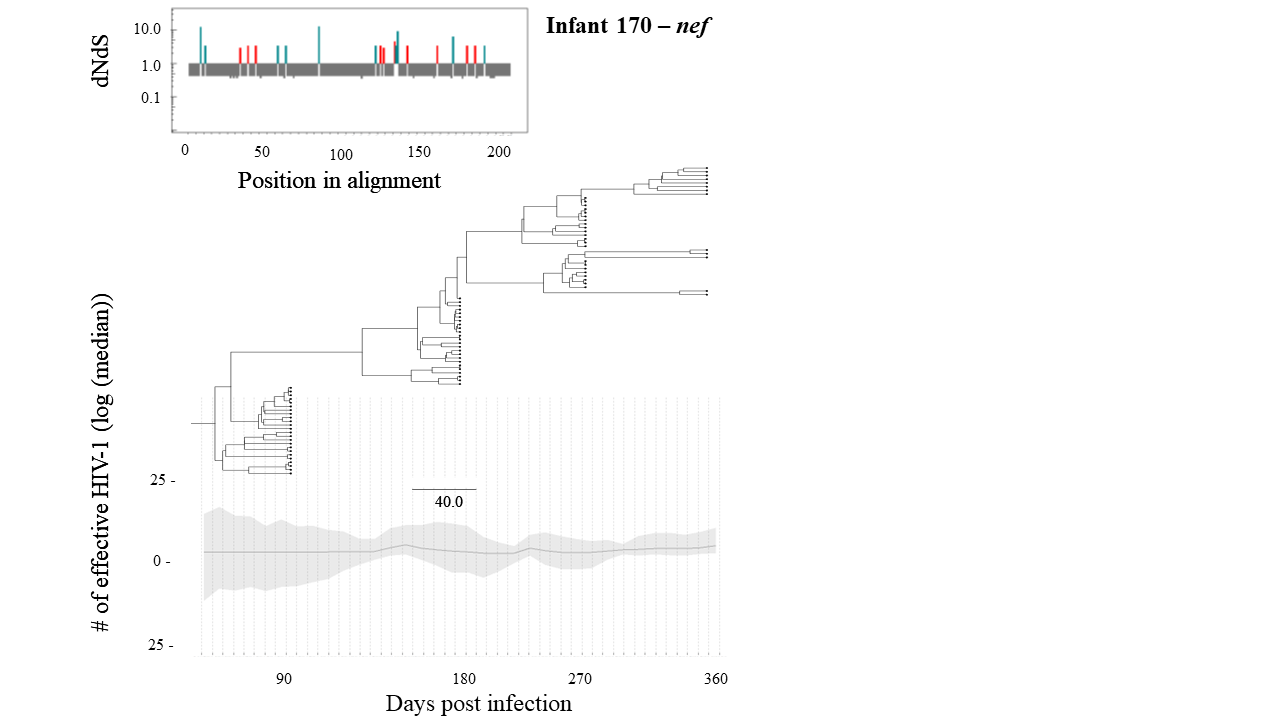

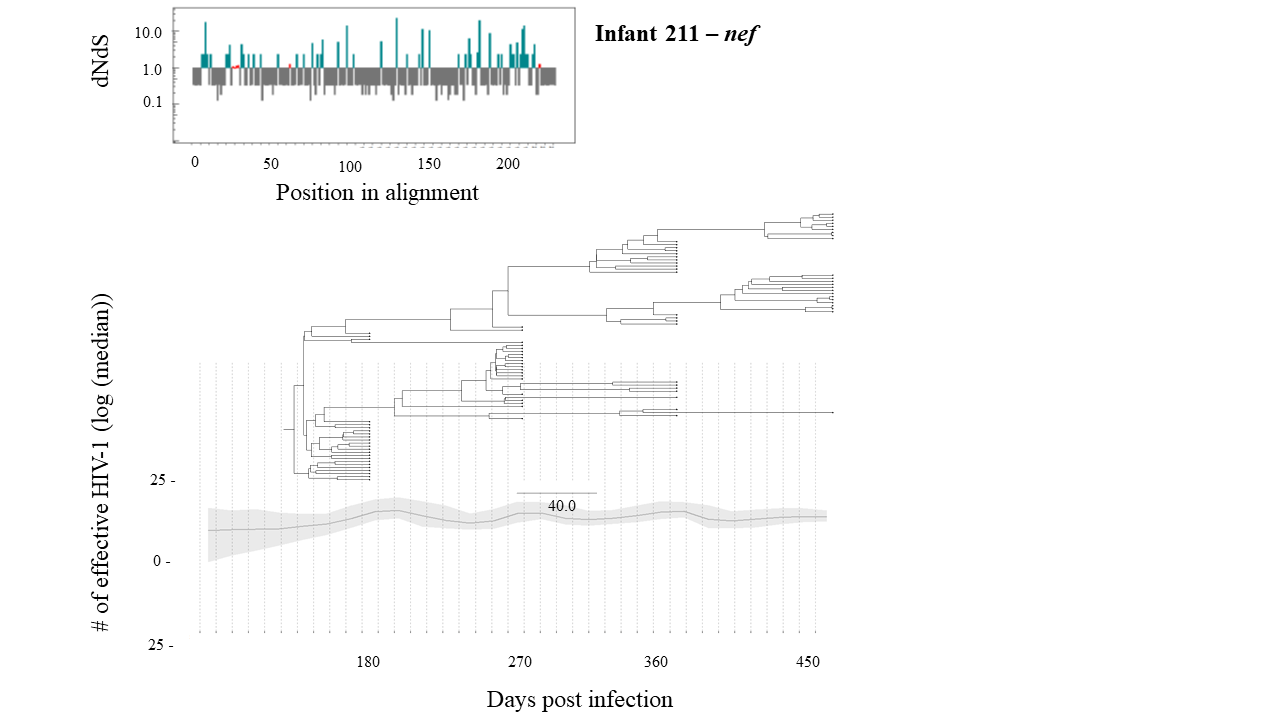

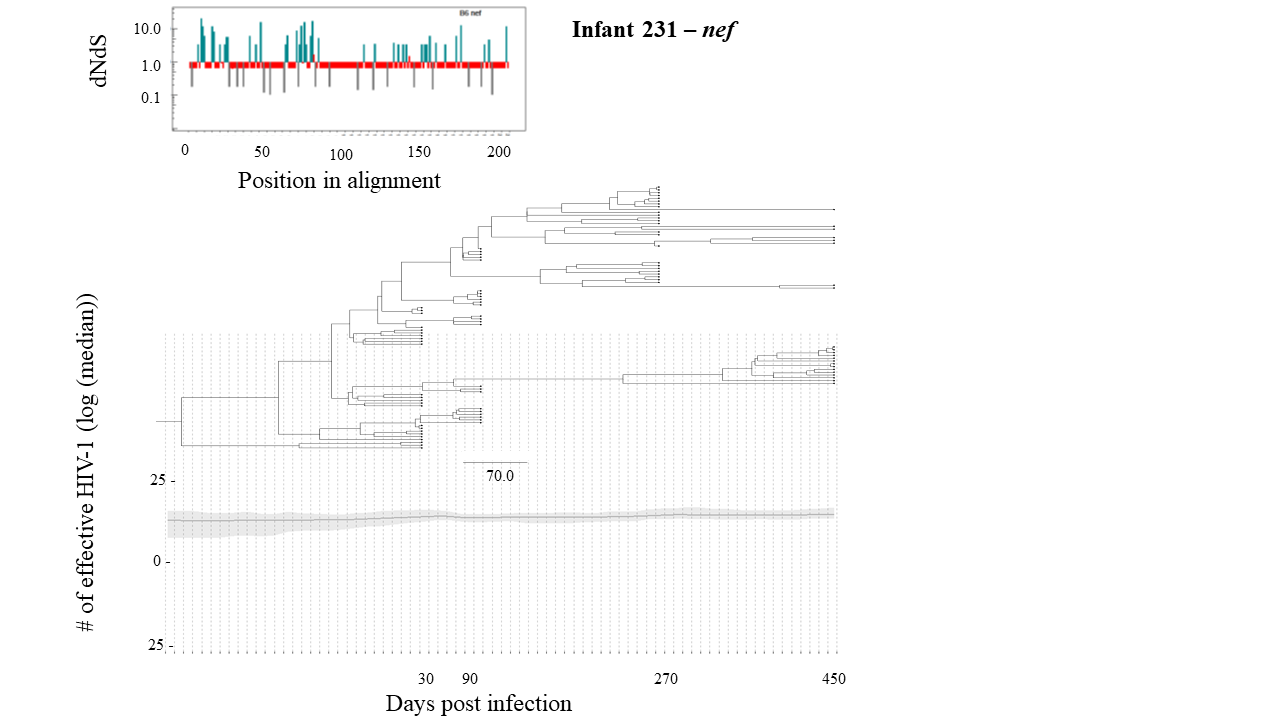

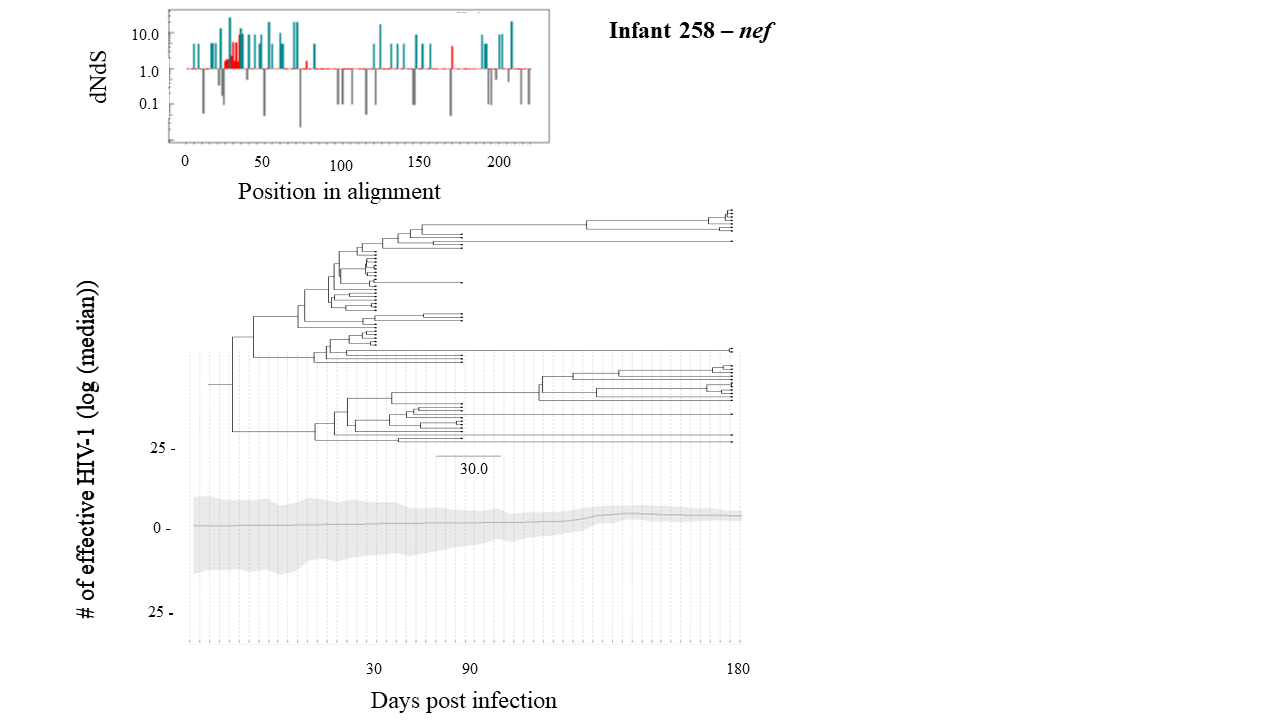

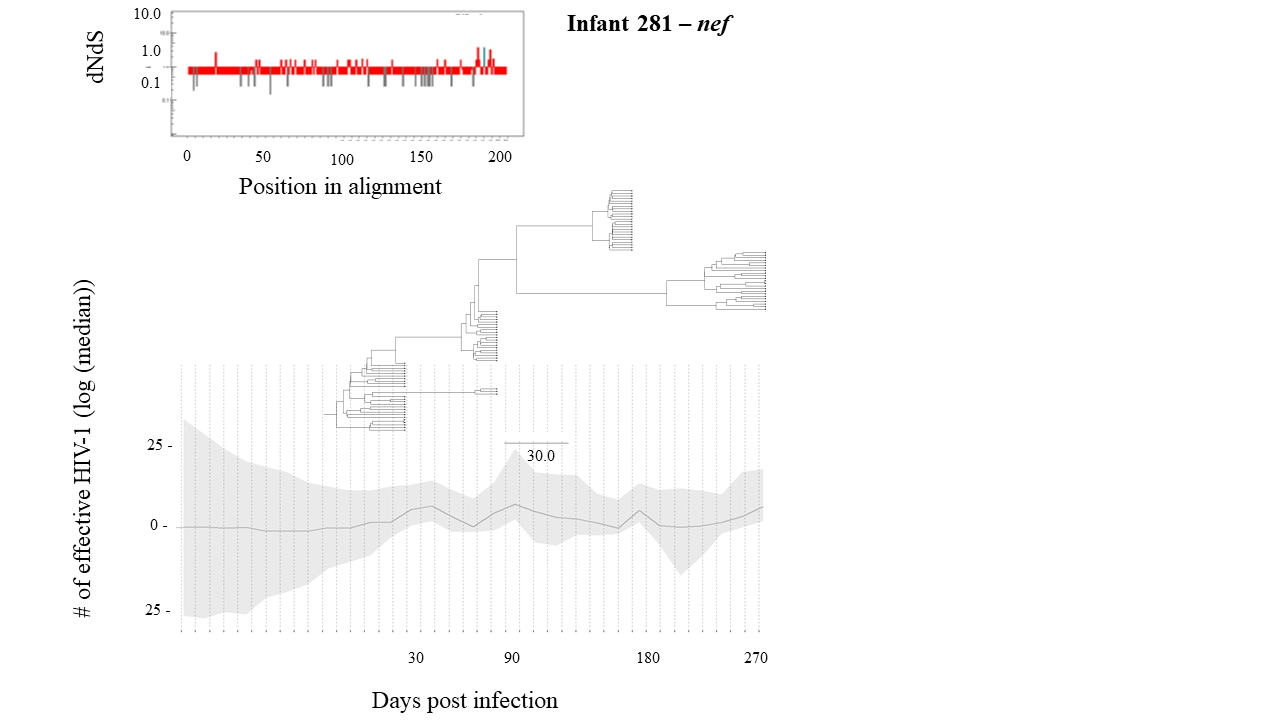

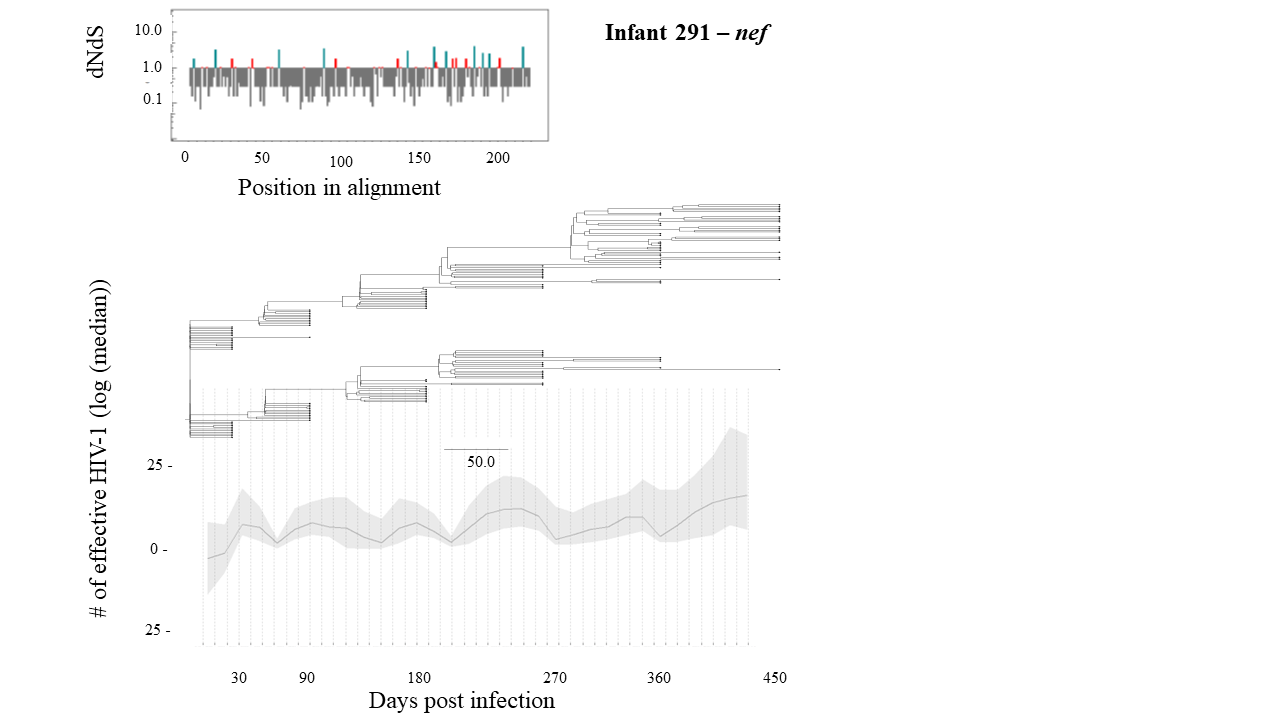

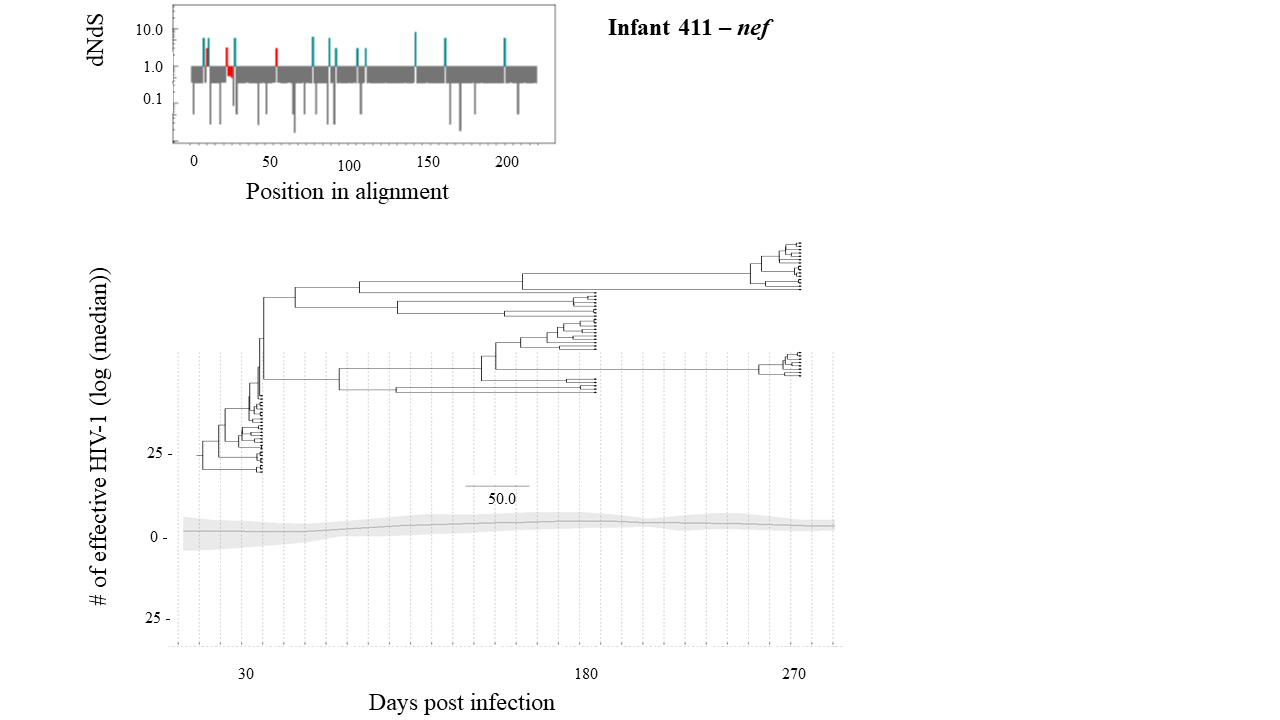

Supplement: Figure S6 — Selective sweeps in gag and nef over time. [file jvi.00072-24-s0002.docx]
