# Supplementary material for: Higher HIV-1 evolutionary rate is associated with cytotoxic T lymphocyte escape mutations in infants
Source: J Virol. 2024 May 30;98(7):e00072-24. doi: 10.1128/jvi.00072-24 (PMC11265422; doi:10.1128/jvi.00072-24)
Supplement: Figures S7 to S9 — Host evolutionary rate and substitution rates. [file jvi.00072-24-s0003.docx]

**HIGHER HIV-1 EVOLUTIONARY RATE IS ASSOCIATED WITH CYTOTOXIC T LYMPHOCYTE ESCAPE MUTATIONS IN INFANTS**

**Authors:** Jamirah Nazziwa (1), Sophie M. Andrews (2), Mimi M. Hou (2), Christian A. W. Bruhn (1), Miguel A. Garcia-Knight (2,3), Jennifer Slyker (4,11), Sarah Hill (5), Barbara Lohman Payne (6,7), Dorothy Mbori-Ngacha (6), Philippe Lemey (8), Grace John-Stewart (4,7,9,10,11), Sarah L. Rowland-Jones (2)*, and Joakim Esbjörnsson (1, 2)§*

*Authors with equal contribution

**Author Affiliations:** (1) Department of Translational Medicine, Lund University, Sweden; (2) Nuffield Department of Clinical Medicine, University of Oxford, UK; (3) Department of Microbiology and Immunology, University of California San Francisco, California, USA; (4) Department of Global Health, University of Washington, Seattle, Washington, United States of America; (5) Department of Pathobiology and Population Sciences, Royal Veterinary College, UK; (6) Department of Paediatrics and Child Health, University of Nairobi, Nairobi, Kenya; (7) Department of Medicine, University of Washington, Seattle, Washington, United States of America; (8) Department of Microbiology, Immunology and Transplantation, Rega Institute, KU Leuven, Leuven, Belgium; (9) Department of Pediatrics, University of Washington, Seattle, Washington, USA; (10) Global Center for Integrated Health of Women, Adolescents and Children (Global WACh), University of Washington, Seattle, Washington, USA; (11) Department of Epidemiology, University of Washington, Seattle, Washington, USA.

**§Corresponding Author:**

Joakim Esbjörnsson

BMC B13

Department of Translational Medicine

Lund University

221 84 Lund, Sweden

Email: [Joakim.esbjornsson@med.lu.se](mailto:Joakim.esbjornsson@med.lu.se)

**SUPPLEMENTARY FIGURES 7-9**

**Figure S7. Infant intra-host evolutionary rate (substitutions per site per year [s/s/y]) in *gag* and *nef*.** (A). Individual infant intra-host evolutionary rate in *gag* and *nef* with 95% highest posterior density (HPD) intervals. A plot indicating the intra-host evolutionary rate for each infant in *gag* (coloured blue) and *nef* (coloured red). The HPD interval is the shortest possible interval with a 95% probability for a parameter of interest (evolutionary rate), given the posterior density function as determined by the likelihood function and the prior distribution. (B) Mean infant intra-host evolutionary rates in *gag* and *nef.* Pirate plots showing the distribution of *gag* (blue) and *nef* (red) intra-host evolutionary rate estimates for each infant (indicated as black dots in each plot). The horizontal line in each pirate plot indicates the mean intra-host evolutionary rate estimate.


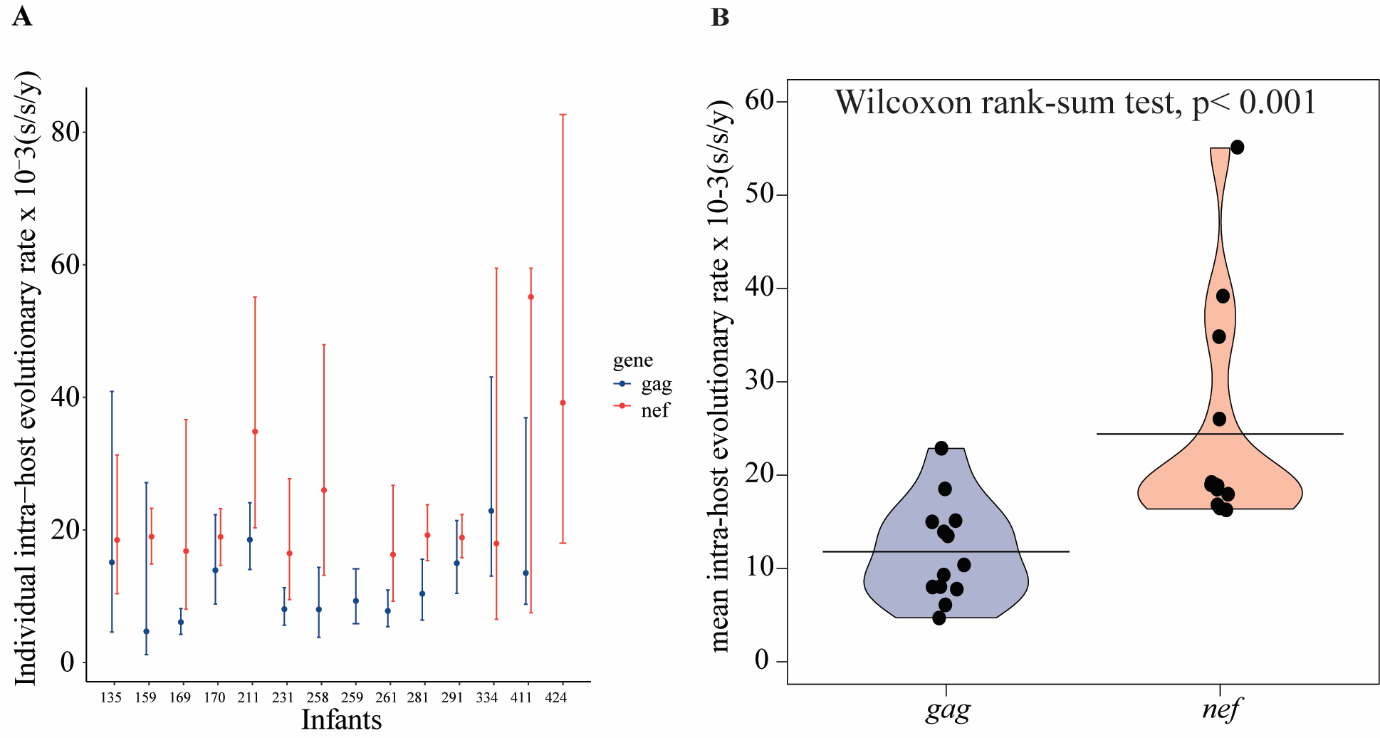


**Figure S8. Infant intra-host evolutionary rate (substitutions per site per year [s/s/y]) in *gag* and *nef* from hierarchical phylogenetic modelling (HPM).** (A). Individual infant intra-host evolutionary rate in *gag* and *nef* with 95% highest posterior density (HPD) intervals using HPM estimates. A plot indicating the intra-host evolutionary rate for each infant in *gag* (coloured blue) and *nef* (coloured red). The HPD interval is the shortest possible interval with a 95% probability for a parameter of interest (evolutionary rate), given the posterior density function as determined by the likelihood function and the prior distribution. (B) Mean HPM infant intra-host evolutionary rates in *gag* and *nef.* Pirate plots showing the distribution of *gag* (blue) and *nef* (red) intra-host evolutionary rate estimates for each infant (indicated as black dots in each plot). The horizontal line in each pirate plot indicates the mean intra-host evolutionary rate estimate.


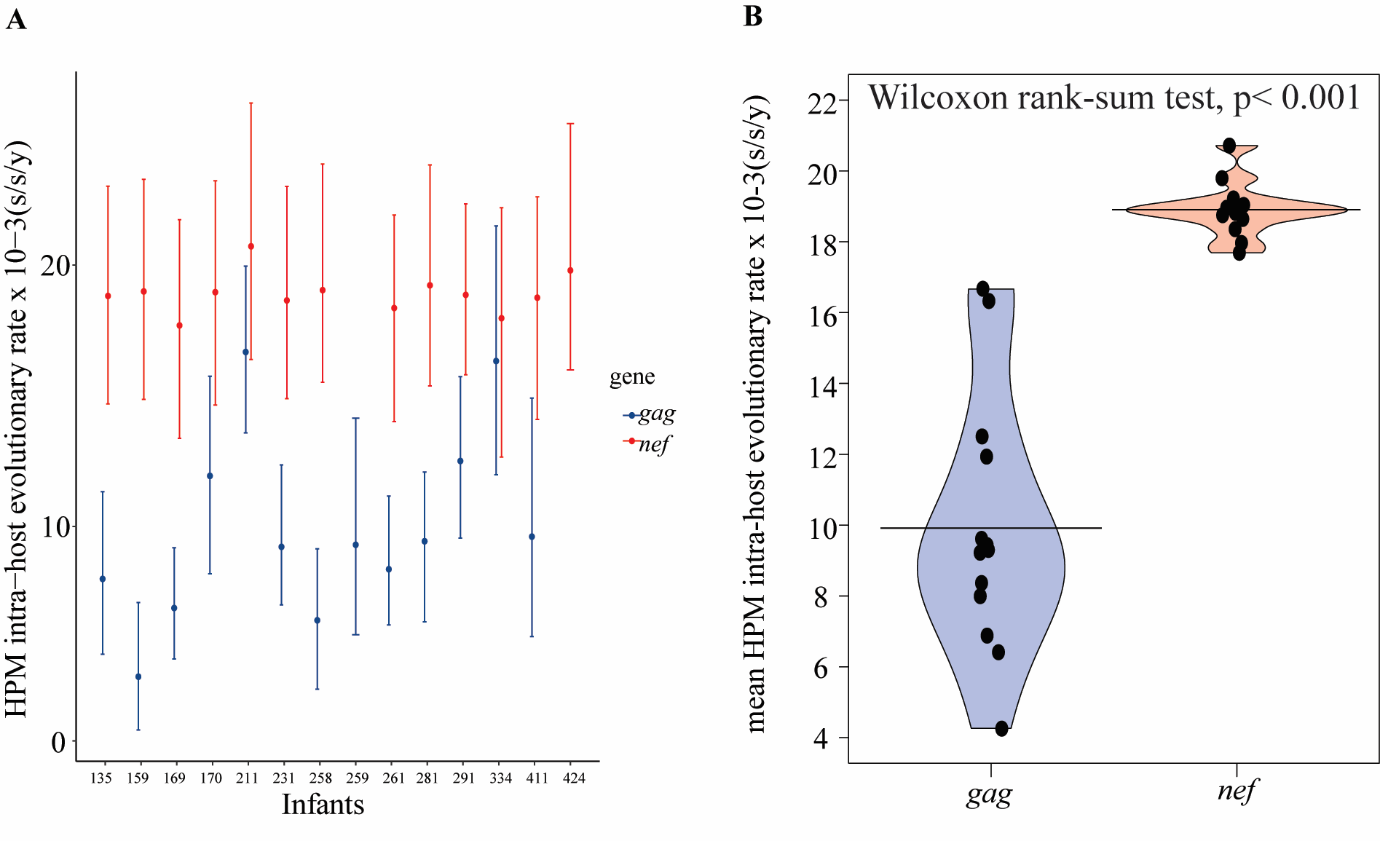


**Figure S9. Infant intra-host nonsynonymous (E[N]) and synonymous (E[S]) substitution rates (substitutions per site per year [s/s/y]) in *Gag*.**

**
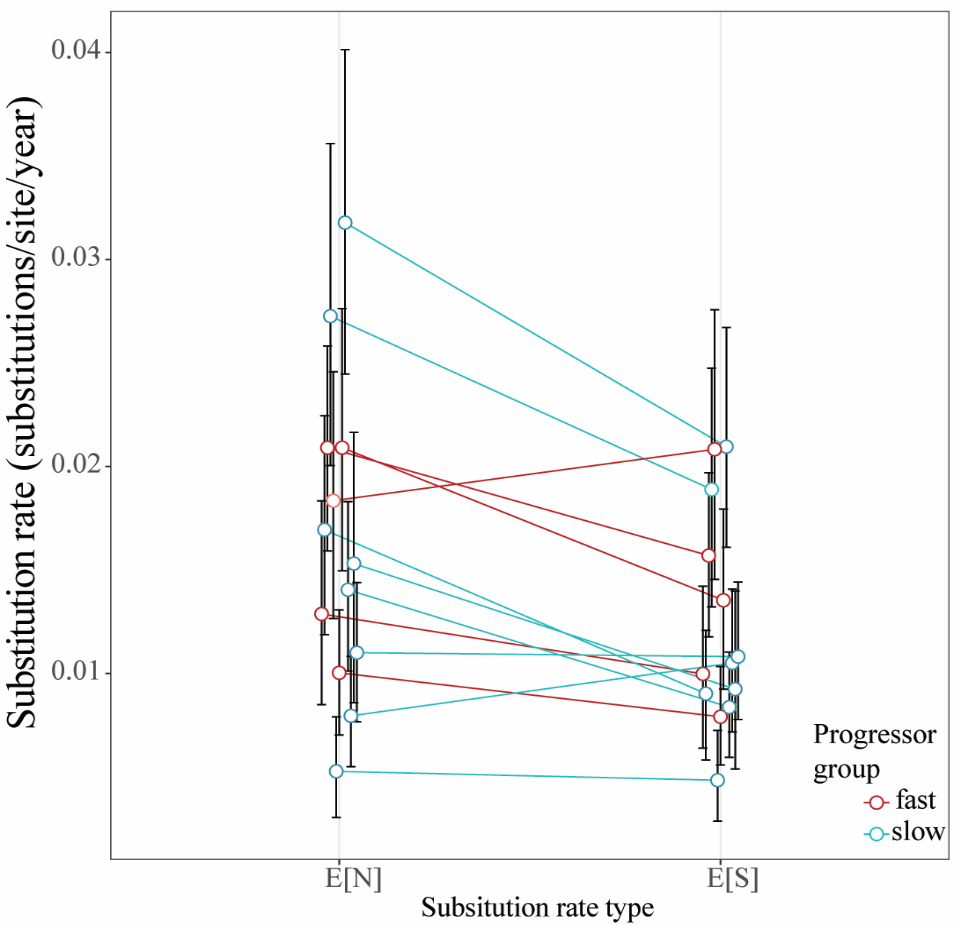
**
