# Supplementary material for: Higher HIV-1 evolutionary rate is associated with cytotoxic T lymphocyte escape mutations in infants
Source: J Virol. 2024 May 30;98(7):e00072-24. doi: 10.1128/jvi.00072-24 (PMC11265422; doi:10.1128/jvi.00072-24)
Supplement: Supplemental tables — Tables S1 to S6. [file jvi.00072-24-s0004.docx]

**HIGHER HIV-1 EVOLUTIONARY RATE IS ASSOCIATED WITH CYTOTOXIC T LYMPHOCYTE ESCAPE MUTATIONS IN INFANTS**

**Authors:** Jamirah Nazziwa (1), Sophie M. Andrews (2), Mimi M. Hou (2), Christian A. W. Bruhn (1), Miguel A. Garcia-Knight (2,3), Jennifer Slyker (4,11), Sarah Hill (5), Barbara Lohman Payne (6,7), Dorothy Mbori-Ngacha (6), Philippe Lemey (8), Grace John-Stewart (4,7,9,10,11), Sarah L. Rowland-Jones (2)*, and Joakim Esbjörnsson (1, 2)§*

*Authors with equal contribution

**Author Affiliations:** (1) Department of Translational Medicine, Lund University, Sweden; (2) Nuffield Department of Clinical Medicine, University of Oxford, UK; (3) Department of Microbiology and Immunology, University of California San Francisco, California, USA; (4) Department of Global Health, University of Washington, Seattle, Washington, United States of America; (5) Department of Pathobiology and Population Sciences, Royal Veterinary College, UK; (6) Department of Paediatrics and Child Health, University of Nairobi, Nairobi, Kenya; (7) Department of Medicine, University of Washington, Seattle, Washington, United States of America; (8) Department of Microbiology, Immunology and Transplantation, Rega Institute, KU Leuven, Leuven, Belgium; (9) Department of Pediatrics, University of Washington, Seattle, Washington, USA; (10) Global Center for Integrated Health of Women, Adolescents and Children (Global WACh), University of Washington, Seattle, Washington, USA; (11) Department of Epidemiology, University of Washington, Seattle, Washington, USA.

**§Corresponding Author:**

Joakim Esbjörnsson

BMC B13

Department of Translational Medicine

Lund University

221 84 Lund, Sweden

Email: [Joakim.esbjornsson@med.lu.se](mailto:Joakim.esbjornsson@med.lu.se)

**SUPPLEMENTARY TABLES**

**Table S1. Infant HIV-1 transmitted/founder virus analysis in *gag* and *nef*.** This table shows infant HIV-1 transmitted/founder virus analysis in *gag*, and *nef*. For each infant with sequences collected at month one, the number of transmitted founder viruses was estimated based on %maximum (max) hamming distance (HD) and the % intra-host sequence diversity. The mode of MTCT of HIV was either *peripartum* (P) or *in utero* (IU) and the first positive PCR or confirmation of HIV-1 infection in infants was observed at month 1 (M1) after birth or week 2 (W2).

| Infant | Mode of transmission  (1st positive PCR) | | Mean intersequence HD | | Max HD | %Max HD | | %Diversity | Result | |
| --- | --- | --- | --- | --- | --- | --- | --- | --- | --- | --- |
| *gag* | | | | | | | | | | |
| 168 | P(M1) | 2.6 | | 9 | | 0.6 | 0.182 [0.177 - 0.187] | | | Infected with a single T/F, % diversity < 0.5 |
| 231 | P(W2) | 3.8 | | 11 | | 0.7 | 0.270 [0.263 - 0.278] | | |  |
| 259 | P(M1) | 2.8 | | 8 | | 0.5 | 0.197 [0.192 - 0.201] | | |  |
| 281 | P(M1) | 2.1 | | 6 | | 0.4 | 0.145 [0.139 - 0.149] | | |  |
| 334 | P(M1) | 4.7 | | 10 | | 0.7 | 0.309 [0.302 - 0.317] | | |  |
| 258 | IU | 11 | | 31 | | 2.1 | 0.671 [0.651 - 0.690] | | | Infected with more than one T/F |
| *nef* | | | | | | | | | | |
| 168 | P(M1) | 0.73 | | 2 | | 0.33 | 0.111 [0.106 - 0.117] | | | Infected with a single T/F, % diversity < 0.5 |
| 291 | P(M1) | 1 | | 4 | | 0.65 | 0.167 [0.155 - 0.167] | | |  |
| 411 | P(M1) | 0.25 | | 2 | | 0.32 | 0.042 [0.035 - 0.042] | | |  |
| 261 | P(M1) | 3 | | 10 | | 1.60 | 0.452 [0.435 - 0.462] | | |  |
| 281 | P(M1) | 2.3 | | 7 | | 1.14 | 0.383 [0.350 - 0.383] | | |  |
| 334 | P(M1) | 2.1 | | 6 | | 0.97 | 0.340 [0.317 - 0.340] | | |  |
| 424 | IU | 2 | | 7 | | 1.13 | 0.338 [0.317 - 0.338] | | |  |
| 231 | P(W2) | 4.9 | | 15 | | 2.44 | 0.720 [0.676 - 0.720] | | | Infected with more than one T/F |
| 258 | IU | 3.4 | | 10 | | 1.58 | 0.628 [0.559 - 0.627] | | |  |

**Table S2. Comparison of phylogenetic tree topologies and seminal methodology by Keele and colleagues in quantification of T/F viruses.**

| **Infant** | ***gag*** | | ***nef*** | |
| --- | --- | --- | --- | --- |
|  | **Keele** | **phylogeny** | **Keele** | **phylogeny** |
| **135** | * | PM | * | * |
| **159** | * | PM | * | * |
| **168** | single | * | single | * |
| **170** | * | * | * | * |
| **211** | * | * | * | * |
| **231** | single | PM | multiple | PM |
| **258** | multiple | PP | multiple | * |
| **259** | single | * | * | * |
| **261** | * | PM | single | PM |
| **281** | single | PM | single | * |
| **291** | * | PM | single | PM |
| **334** | single | PM | single | PM |
| **411** | * | PP | single | * |
| **424** | NA | NA | single | PM |

***gag***

|  |  | **TF = 1** | **TF > 1** |
| --- | --- | --- | --- |
| **Phylogeny: N = 9** | **PM** | **7** | **0** |
|  | **PP** | **0** | **2** |
| **Keele methodology: N = 6** | **Keele** | **5** | **1** |

***nef***

|  |  | **TF = 1** | **TF > 1** |
| --- | --- | --- | --- |
| **Phylogeny: N = 5** | **PM** | **5** | **0** |
|  | **PP** |  | **0** |
| **Keele methodology: N = 9** | **Keele** | **7** | **2** |

**Table S3. Amino acid signatures (non-synonymous AA changes) in Gag.** Table indicating amino acid signatures (non-synonymous AA changes) in Gag. Amino acid alignments from each patient were compared between time-points as the infection progressed. Time-points where no clonal sequences were collected are marked with a ‘-‘. No amino acid changes in the alignments across time-point comparisons are marked with ‘*‘. Amino acid changes located at epitope positions are underlined whereas amino acid changes with ‘*’ indicate a change from hydrophilic to neutral amino acid.

| PID | Number of clonal sequences per infant | | | | | | % of non-synonymous changes per infant per time point comparison | | | | | |
| --- | --- | --- | --- | --- | --- | --- | --- | --- | --- | --- | --- | --- |
|  | **M1** | **M3** | **M6** | **M9** | **M12** | **M15** | **M1** | **M3** | **M6** | **M9** | **M12** | **M15** |
| 135 | - | - | 20 | 24 | 10 | - | - | - | - | * | K18R - 70% R30S* - 10% R30T* - 70% T149N^ƨ^ - 70% | - |
| 159 | - | 24 | - | - | - | 20 | - | * | - | - | NA | - |
| 168 | 17 | 22 | 24 | - | 21 | - | * | NA | NA | - | E119G* - 57% E119A* - 33% | - |
| 170 | - | - | 19 | 22 | 24 | - | - | - | * | S496L^ϫ^ - 50% | K76R - 80% K331R - 88% | - |
| 211 | - | 21 | 22 | 23 | 19 | 23 | - | * | P382S - 50% L382P* - 30% | C87W - 97% E90K - 96% Q216H* - 96% | G179A - 78% I188V - 79% | K28R - 83%K114Q - 83% T179A - 96% L212V - 61% S382P - 35% |
| 231 | 23 | 24 | - | 21 | - | 21 | * | N253T*- 96% S487L^ϫ^ - 67% | - | NA | - | I391N^ƨ^ - 100% L487V - 38% |
| 258 | 20 | - | 23 | - | 24 | - | * | - | T54A - 6% L64I - 61% S66P - 61% | - | R18K*- 96% A54T - 54% I64L - 87% P66S - 87% | - |
| 259 | 23 | - | - | - | 21 | - | * | - | - | - | A15K^ƨ^ - 81% N26K - 62% M31L - 57% T84V^ϫ^ - 96% | - |
| 261 | - | 22 | 24 | 22 | - | 23 | - | * | NA | Q69R - 46% |  | V215A^ƨ^ - 48% E474K - 96% |
| 281 | 24 | 24 | 20 | 24 | - | - | * | NA | S9R^ƨ^ -75% I370V - 70% | R9S^ƨ^ - 46% Q30R - 54% F382I - 42% | - | - |
| 291 | - | - | 24 | 24 | 21 | 22 | - | - | * | N146S* - 54% A336G - 79% | E42D^ƨ^ - 81% E62G - 76% | S332T - 96% |
| 334 | 21 | 18 | 21 | 23 | - | - | * | R91K - 78% V143A - 94% | A143V - 95% H144Y - 100% I147M - 100% | I436L - 87% | - | - |
| 411 | - | 22 | 22 | 21 | - | - | - | * | D42E - 68% S49G - 63% P66S - 73% S67A - 62% V75I - 76% R123G - 86% T373A - 82% C461Y - 86% L484S - 91% | E42D - 62% G49S - 62% S66P - 62% S67A - 62% V75I - 76% | - | - |

.

**Table S4.** **Infants HLA types included in sub-analysis.**

| HLA type | Patient ID | | | | | |
| --- | --- | --- | --- | --- | --- | --- |
| A29 | 159 | 168 | 261 | 291 | 334 |  |
| A30 | 159 | 170 | 211 | 258 | 411 |  |
| A74 | 259 | 281 | 291 |  |  |  |
| A6802 | 135 | 211 | 424 |  |  |  |
| B15 | 168 | 258 | 281 | 291 | 334 | 411 |
| B4201/2 | 170 | 211 | 291 |  |  |  |
| C2 | 168 | 281 | 291 | 334 |  |  |
| C4 | 135 | 168 | 231 |  |  |  |
| C6 | 159 | 261 | 334 |  |  |  |
| C7 | 170 | 211 | 259 | 261 | 424 |  |
| C17 | 170 | 211 | 258 | 291 | 411 |  |

**Table S5A: *Gag* dN/dS ratios for amino acid positions within HLA-restricted epitopes.**

| **Infant ID** | **Amino acid positions** | **Gag CTL epitopes** | **dN/dS ratio per epitope position** | | | | | | | | | | | |
| --- | --- | --- | --- | --- | --- | --- | --- | --- | --- | --- | --- | --- | --- | --- |
| **135** | 76-86 | kSLfNaVAvLY | 3.3 | 0.5 | 0.5 | 0.5 | 0.5 | 0.5 | 0.1 | 0.5 | 1.9 | 0.5 | 0.5 |  |
|  | 180-188 | TPQDLNmML | 1.9 | 0.5 | 0.5 | 0.5 | 0.1 | 0.5 | 0.5 | 0.5 | 0.1 |  |  |  |
|  | 272-285 | YSPvSILDI | 0.1 | 0.5 | 1.9 | 0.5 | 0.5 | 0.5 | 0.5 | 0.5 | 0.5 |  |  |  |
| 168 | 28-36 | KYRLKHlVW | 0.4 | 0.1 | 0.1 | 0.4 | 0.4 | 0.4 | 0.4 | 0.4 | 0.4 |  |  |  |
| 170 | 76-86 | kSLYNTVATLY | 11.3 | 0.4 | 0.4 | 0.4 | 2.0 | 0.4 | 0.4 | 2.0 | 0.4 | 2.0 | 0.4 |  |
| 211 | 76-86 | kSLYNTVAvLY | 0.2 | 0.2 | 4.1 | 0.2 | 0.2 | 4.1 | 0.2 | 0.1 | 0.2 | 2.1 | 13.8 |  |
|  | 180-188 | TPgDLNTML | 0.1 | 0.2 | 15.2 | 0.6 | 0.0 | 0.1 | 0.2 | 0.2 | 0.2 |  |  |  |
|  | 385-393 | GsRriiKCF | 4.1 | 11.6 | 21.8 | 7.4 | 21.2 | 12.1 | 0.0 | 0.2 | 8.8 |  |  |  |
| **231** | 28-36 | KYRLKHlVW | 2.4 | 0.5 | 0.5 | 0.5 | 0.1 | 0.5 | 0.5 | 0.5 | 0.5 |  |  |  |
| **258** | 18-26 | KIRLRPGGK | 17.1 | 0.0 | 0.4 | 0.4 | 0.7 | 0.4 | 0.4 | 0.4 | 0.4 |  |  |  |
|  | 180-188 | TPQDLNmML | 0.4 | 0.4 | 0.4 | 0.4 | 0.4 | 0.4 | 0.4 | 0.4 | 0.0 |  |  |  |
| 259 | 28-36 | KYRmKHlVW | 0.5 | 0.5 | 4.6 | 17.9 | 0.5 | 0.5 | 0.5 | 0.5 | 0.5 |  |  |  |
|  | 180-188 | TPQDLNmML | 0.5 | 0.5 | 0.5 | 0.5 | 0.5 | 0.5 | 0.5 | 0.5 | 0.1 |  |  |  |
| **261** | 180-188 | TPQDLNmML | 0.4 | 0.1 | 0.4 | 0.4 | 0.4 | 0.1 | 0.4 | 0.4 | 0.1 |  |  |  |
| **291** | 180-188 | TPQDLNmML | 0.4 | 0.4 | 1.6 | 0.4 | 0.1 | 0.1 | 0.4 | 0.4 | 0.1 |  |  |  |
| **334** | 145-155 | QAmSPRTLNAW | 0.8 | 0.8 | 1.8 | 0.8 | 0.8 | 0.8 | 0.8 | 0.8 | 1.8 | 0.8 | 0.8 |  |
|  | 167-175 | EVIPMFtAL | 0.1 | 0.8 | 0.8 | 0.8 | 0.8 | 1.8 | 1.8 | 0.8 | 0.8 |  |  |  |
|  | 294-304 | RDYVDRFFKiL | 0.8 | 0.8 | 0.8 | 0.8 | 0.8 | 0.2 | 0.2 | 0.8 | 0.8 | 0.8 | 0.8 |  |
|  | 306-317 | AEQAtQEVKgWMT | 0.8 | 0.8 | 0.8 | 1.8 | 0.8 | 0.8 | 1.8 | 0.8 | 1.8 | 0.8 | 0.8 | 0.8 |
|  | 429-437 | RQANFLGKI | 0.2 | 4.9 | 0.8 | 0.2 | 0.2 | 0.8 | 1.8 | 0.8 | 5.5 |  |  |  |
| **411** | 18-26 | KIRLRPGGK | 6.2 | 0.5 | 0.5 | 3.4 | 0.1 | 0.5 | 0.5 | 3.3 | 0.5 |  |  |  |
|  | 76-86 | kSLfNaVAvLf | 0.5 | 0.5 | 0.5 | 0.5 | 0.5 | 6.2 | 6.2 | 0.1 | 0.5 | 0.5 | 0.5 |  |
|  | 180-188 | TPQDLNmML | 0.5 | 0.5 | 0.5 | 0.5 | 0.5 | 0.5 | 0.6 | 0.6 | 0.0 |  |  |  |

**Table S5B: *Nef* dN/dS ratios for amino acid positions within HLA-restricted epitopes.**

| **Infant ID** | **Amino acid positions** | **Nef CTL epitopes** | **dN/dS ratio per epitope position** | | | | | | | | | | | |
| --- | --- | --- | --- | --- | --- | --- | --- | --- | --- | --- | --- | --- | --- | --- |
| 170 | 77-85 | sPMTYKAAi | 0.4 | 0.4 | 0.4 | 0.4 | 0.4 | 0.4 | 0.4 | 0.4 | 12.4 |  |  |  |
| 211 | 71-79 | kPQVPLRPM | 0.4 | 0.4 | 0.4 | 0.4 | 0.4 | 0.4 | 0.4 | 0.4 | 0.4 |  |  |  |
|  | 77-85 | RPMTYKAAV | 0.2 | 0.3 | 0.3 | 0.3 | 0.3 | 0.3 | 0.2 | 0.3 | 5.0 |  |  |  |
| **334** | 120-128 | YFPDWQNYT | 1.0 | 0.7 | 0.7 | 0.7 | 0.7 | 1.0 | 0.3 | 0.7 | 0.7 |  |  |  |
| **424** | 83-91 | gAlDLSHFL | 0.7 | 0.7 | 2.2 | 0.7 | 0.3 | 0.7 | 0.7 | 2 | 0.7 |  |  |  |

Infants with mother sequences included in the analysis are denoted in bold.

Amino acid positions indicated in lower case denote differences in infant sequences (subtype A1) compared to the HXB2 reference, as documented in the LANL database.

Epitopes highlighted in yellow indicate those epitopes that remained unchanged or consistent within both the mother and infant across all time points.

Epitopes highlighted in red indicate epitopes that exhibited changes specifically within the infant after six months post-infection, without corresponding observations in the mother or the transmitted founder virus population in the infant.

The dN/dS ratios highlighted in red represent the amino acid positions that were under positive selection (dN/dS ratio >1).

**Table S6. CTL escape variants and other variants observed in both Gag and Nef infant and mother sequences**

| Infant+ Mother | Infant HLA | **Gag** | | | | | **Nef** | | | |
| --- | --- | --- | --- | --- | --- | --- | --- | --- | --- | --- |
|  |  | CTL escape variants | Variants lost or maintained over time | Other observed variants | Variants lost or maintained over time | ELISPOT results | CTL escape variants | Time points/variants lost or maintained over time | Other observed variants | Variants lost or maintained over time |
| 135 +  Mother | A*32 + 6802 | 3: |  | 5: |  |  |  |  | 2: |  |
|  | B*39 + 4501 | 76 – 88 | * | 28 – 36 | Maintained M6, 9, 12 | - |  |  | 71 – 79 | 58% M9  41% M12 |
|  | Cw*4 + 12 | 180 – 188 | * | 84 – 92 | * | - |  |  | 105 -115 | * |
|  |  | 272 – 285 | * | 160 – 168 | 25% at M9 | - |  |  |  |  |
|  |  |  |  | 294 – 304 | * | - |  |  |  |  |
|  |  |  |  | 308 – 316 | * | Wild type recognised at M3 |  |  |  |  |
| 159 +  Mother | A*29 + 30 |  |  |  |  |  |  |  |  |  |
|  | B*4501 |  |  |  |  |  |  |  |  |  |
|  | Cw*6 |  |  |  |  |  |  |  |  |  |
| 168 | A*29 + 30 | 1: |  | 2: |  |  |  |  | 1: |  |
|  | B*15 + 5801 | 28 – 36 | All months | 274 – 282 | * |  |  |  | 80-87 | Lost at M12 |
|  | Cw*2 + 4 |  |  | 295 – 304 | * |  |  |  |  |  |
| 170 | A*30 + 3402 | 1: |  | 1: |  |  | 1: |  | 3: |  |
|  | B*42 + 57 | 76 – 86 | Decreased from M6 100% - M12 20 % | 20-29 | * |  | 77-85 | Decreased from M3 | 105-114 | * |
|  | Cw*7 + 17 |  |  |  |  |  |  |  | 128-137 | * |
| 211 | A*30 + 6802 | 3: |  | 1: |  |  | 2: |  |  |  |
|  | B*42 | 76 – 86 | * | 83 – 91 | * |  | 71-79 | Decreased from M6 20% - M15 87 % |  |  |
|  | Cw*7 + 17 | 180 – 188 | * |  |  |  | 77 – 85 | * |  |  |
|  |  | 385-393 | 30% m15 |  |  |  |  |  |  |  |
| 231 +  Mother | A*26 + 34 | 1: |  | 1: |  |  |  |  |  |  |
|  | B*35 + 53 | 28 – 36 | * | 294 – 304 | * |  |  |  |  |  |
|  | Cw*4 |  |  |  |  |  |  |  |  |  |
|  |  |  |  |  |  |  |  |  |  |  |
| 258 + | A*3 + 30 | 1: |  |  |  |  |  |  | 1: |  |
| Mother | B*15 + 7301 | 18 – 26 | Lost at m12 |  |  |  |  |  | 128-137 | * |
|  | Cw* 15 + 17 | 180 – 188 | * |  |  |  |  |  |  |  |
| 259 | A*23 + 74 | 1: |  |  |  |  |  |  |  |  |
|  | B*58 + 4501 | 28 – 36 | * |  |  |  |  |  |  |  |
|  | Cw*7 | 180 – 188 | * |  |  |  |  |  |  |  |
| 261 +  Mother | A*2 + 74 | 1: |  | 1: |  |  |  |  | 1: |  |
|  | B*5802 + 35 | 180 – 188 | * | 433- 442 | Only infant |  |  |  | 134-143 | Only m9 and m15 |
|  | Cw*6 + 7 |  |  |  |  |  |  |  |  |  |
| 281 +  Mother ^a^ | A*23 + 74 |  |  |  |  |  |  |  | 2 |  |
|  | B*15 |  |  |  |  |  |  |  | 127-135 | * |
|  | Cw*2 |  |  |  |  |  |  |  | 77 -85 | * |
| 291 +  Mother^a^ | A*29 + 74 | 1: |  |  |  |  |  |  | 1: |  |
|  | B*4201 + 15 | 180 – 188 | * |  |  |  |  |  | 77-85 | Persisted over time |
|  | Cw*2 + 17 |  |  |  |  |  |  |  |  |  |
| 334 +  Mother | A*29 + 26 | 5: |  |  |  |  | 1: |  |  |  |
|  |  | 167-175 | * |  |  |  | 120-128 | Found in mother, lost after m3 |  |  |
|  |  | 145 – 155 | Appeared at m6, m9 but not at m1, m3 |  |  |  |  |  |  |  |
|  | B*13 + 15 | 294 – 304 | * |  |  |  |  |  |  |  |
|  |  | 306 -317 | * |  |  |  |  |  |  |  |
|  | Cw*2 + 6 | 429 – 437 | Increased at M9 |  |  |  |  |  |  |  |
| 411 + | A*30 | 3 |  |  |  |  |  |  |  |  |
| mother^a^ | B*15+ 42 | 180-188 | * |  |  |  |  |  |  |  |
|  | Cw*14 + 17 | 76-86 | * |  |  |  |  |  |  |  |
|  |  | 20-29 | * more than 85% at m1,3 |  |  |  |  |  |  |  |
| 424 | A*2 + 6802 |  |  |  |  |  | 1 |  | 1 |  |
|  | B*51 + 8 |  |  |  |  |  | 83-91 | Decreased to 50% m6 | 190-198 | * |
|  | Cw*7 + 16 |  |  |  |  |  |  |  |  |  |
|  |  |  |  |  |  |  |  |  |  |  |

^a^No mother sequences in Nef.

*transmitted from the mother and/or the same variant over follow-up time.

Not all infants had mother sequences available. HLA restricted escape variants are indicated with the Gag HXB2 position. Values given as % indicate the percentage of sequences with the specified CTL variant.
